# A new weapon: the application of tumor vaccines based on extracellular exosomal heat shock proteins in immunotherapy

**DOI:** 10.3389/fimmu.2025.1510650

**Published:** 2025-01-22

**Authors:** Kexin Yi, Chengpeng Sun, Yalin Yuan, Zhaowei Luo, Hongliang Luo, Yunhe Xie

**Affiliations:** ^1^ The Second Clinical Medical College, Nanchang University, Nanchang, China; ^2^ Huankui Academy, Jiangxi Medical College, Nanchang University, Nanchang, Jiangxi, China; ^3^ Department of General Surgery, The Second Affiliated Hospital, Jiangxi Medical College, Nanchang University, Nanchang, Jiangxi, China; ^4^ Department of General Surgery, Jiujiang Hospital of Traditional Chinese Medicine, Jiujiang, Jiangxi, China

**Keywords:** heat shock proteins, exosomes, immunotherapy, tumor vaccines, tumor microenvironment

## Abstract

Despite the significant advancements in cancer research, innovative approaches are still needed to reduce tumor incidence, progression, and dissemination, as well as for prolonging patient survival. Currently, the development of cancer vaccines is gaining attention as a novel preventative and therapeutic strategy. Although the concept of cancer vaccination is not new, a limited number of vaccines have received approval for tumor therapy. Heat shock protein (HSP)-based vaccination represents a promising strategy that harnesses specific tumor antigens to activate immune responses. Exosomes (Exs) are highly heterogeneous bilayer vesicles capable of transporting various types of molecules through extracellular space. Compared with conventional anticancer drugs, exosomes exhibit low toxicity and good biocompatibility, and they can stimulate the immune system either directly or indirectly. Ex-based vaccines may elicit an antitumor immune response that generates memory cells capable of recognizing cancer antigens, thereby inhibiting disease progression. This paper reviews the potential applications of HSPs and exosomes in the prevention and treatment of solid tumors. Finally, we discuss the advantages of the extracellular exosomal heat shock protein (HSP-Ex^1^) vaccine and future research directions aimed at optimizing heat shock protein-based cancer immunotherapy strategies.

## 1 Introduction

With the aging and rapid growth of the global population, cancer has become a major health threat to all of mankind in the twenty-first century. According to the analysis of relevant population data from 2005–2020, the total number of cancer-related deaths increased by 21.6%, and malignant neoplasms are now the leading factor in disease-related deaths (1, 2). HSPs constitute a large family of proteins that are involved in protein folding and maturation; moreover, they are usually produced when the body is induced by heat shock or stressors. HSPs protect cells in extreme states (low temperature, hypoxia, anoxia, and heavy metals), maintain protein structure and function, and degrade unrepairable proteins (3). Notably, the growth of cancer cells requires large amounts of proteins to maintain their metabolic functions. The stress-induced expression of HSPs in cancer cells may play a pivotal role in maintaining protein homeostasis and ensuring the proper synthesis of proteins. HSPs are responsible for forming, assembling, and maturing macromolecular structures in cancer cells (4). HSP expression has been reported to be significantly elevated in many cancers; for example, high levels of heat shock proteins affect six phases of “cancer hallmarks” via the activation or inhibition of cellular pathways, including 1) sustained proliferative signaling, 2) evasion of growth inhibitory factors, 3) resistance to programmed cell death, 4) infinite replication, 5) induction of aberrant angiogenesis, and 6) activation of invasion and metastasis (5). In addition, the overexpression of heat shock proteins is strongly associated with treatment resistance and poor survival (6–8).

Prior to the 21st century, the main treatments for cancer included surgical resection, interventional radiotherapy and chemotherapy (9). However, the abovementioned treatments inevitably damage the patient’s own immune system and are associated with significant toxic side effects and immune-related complications. Chemotherapy and intervention may lead to the destruction of normal immune cells and functional cells, thus affecting both the prognosis of patients and their survival rates. Therefore, there is an urgent need to identify safer and more effective anticancer methods. Immunotherapy has ushered in a new era of tumor treatment, and tumor vaccines are beginning to publicly emerge and are becoming more well-known (10). Tumors usually consist of fragments containing specific tumor cells. Tumor vaccines are clinically available for treatment and prevention. Prostate and lung cancer vaccines are used to treat prostate cancer and small cell carcinoma of the lung, respectively. HPV vaccines are used to prevent cervical cancer, and hepatitis B vaccines are used to prevent HBV (hepatitis B virus) infection, as well as reduce the incidence of cervical and liver cancer (11–14). HSPs can form tumor-vaccine complexes in various forms, which interact with receptors on antigen-presenting cells, promote dendritic cell maturation, lead to the upregulation of MHC-I and MHC-II, and induce immunomodulatory effects on T-cells. Compared with traditional tumor vaccines, heat shock protein vaccines have more effective immune advantages in tumor therapy and have better application prospects (15). Heat shock proteins are important biomarkers in some tissues and indicate the degree of differentiation and aggressiveness of certain cancers with some specificity (16–18). In addition, tremendous progress has been made in identifying heat shock proteins as biomarkers for potential cancer diagnosis and treatment (19, 20).

This review summarizes the molecular regulatory mechanisms of the interactions between HSPs and HSP-Exs in terms of tumor growth, proliferation, metastasis, apoptosis and the tumor immune microenvironment. Recent advances in the use of heat shock proteins as tumor vaccines in tumor therapy are also further discussed to provide new ideas for cancer treatment. In addition, this study highlights the regulatory role of HSP-Exs in the tumor immune microenvironment (TIME) and explores the potential advantages and limitations of using HSP-Exs as next-generation tumor vaccines in the context of current clinical cancer therapies.

## 2 Roles of HSPs in cancer development and progression

HSPs are a diverse family of proteins that are widely found in prokaryotes and eukaryotes; moreover, they are highly conserved. As a family of molecular chaperones that are conserved during evolution, HSPs play key roles in cell survival and cell replication. HSPs are overexpressed in most human cancers and are involved in the development of many cancers, including cancerous activities such as cell proliferation, migration, invasion, angiogenesis, metastasis, and apoptosis resistance. HSPs are classified into several isoforms based on their molecular weights, including HSP110, HSP90, HSP70, HSP60, HSP40, and a number of small HSPs, which play crucial roles in regulating the cell cycle and cell apoptosis. Studies to date have identified the unique functions of multiple HSPs that are involved in protein synthesis and translation (21). The relationships among HSP subtypes and their localization in tumor regulation are summarized in **Table 1**.

**Table 1:** The localizations of various types of HSPs and the mechanisms by which they are associated with different tumors.

Subtypes	Intracellular localization	Tumor	Regulatory pathways	Reference
HSP90	endoplasmic reticulum, mitochondria	Colorectal cancer	HSP90 promotes colon cancer invasion and metastasis as well as epithelial-mesenchymal transition through activation of cytokines HIF-1α and NF-κB.	(22)
		Thyroid cancer	The overexpression of HSP90 is closely related to the malignancy of the tumor and the survival and prognosis of patients.	(23)
		Leukemia	Inhibition of HSP90 depletes TrkA and its pro-growth and pro-survival signals in myeloid leukemia cells, thereby inhibiting tumor growth.	(24)
		Ovarian cancer	Aberrant expression of HSP90 in ovarian cysts may inhibit vesicle apoptosis and delay vesicle degeneration	(25)
HSP70	Nucleus, cytoplasmic lysate mitochondria, endoplasmic reticulum	Gastric adenocarcinoma	The interaction between HSP70 and P53 promotes tumor growth, progression, and invasion and is associated with poor patient prognosis.	(26)
		Osteosarcoma	HSP70 can be used as a marker for conventional and low-grade central osteosarcoma.	(27)
			HSP72 is involved in the interaction between T lymphocytes and osteosarcoma cells in specific osteosarcoma groups. Induction of HSP72 in osteosarcoma may potentiate the immune response and promote tumor rejection.	(28)
		Bladder cancer	HSP70 expression correlates with bladder cancer staging	(29, 30)
		Thyroid cancer	Upregulation of HSP70 helps tumors evade antitumor therapy by activating the RAF/MEK/ERK signaling pathway. In addition, HSP70 promotes cytoplasmic segregation of TP53 and negatively regulates its tumor suppressor function.	(23)
HSP60	Mitochondria The outer compartments of the mitochondrion include the cytoplasm, the outer surface of the mitochondrion, intracellular vesicles, the nucleus, the extracellular space, and the circulation.	Renal cell carcinoma	Downregulation of HSP60 with HSP60 knockdown drives metabolic reprogramming in renal cell carcinoma, a process that promotes tumor progression and enhances mitochondria-dependent biosynthesis.	(31)
		Bronchial lung cancer	Bronchial biopsy shows substantial downregulation of HSP60.	(32)
		Adenocarcinoma of the Lung	HSP60 showed strong positive staining and its expression level was not only correlated with TNM staging but also significantly correlated with prognosis.	(32)

### 2.1 HSP90

HSP90 has a molecular weight of approximately 90 kDa, and its structure usually contains α-α or β-β homodimers (33). HSP90 contains three conserved structural domains: the N-terminal domain (NTD), the middle domain (MD), and the C-terminal domain (CTD) (34).

Notably, the N-terminal structural domain binds to ATP and is also known as the nucleotide-binding domain. Unlike prokaryotic cells, there is a short dynamic region of HSP90 in eukaryotic cells that connects the NTD to the MD, which can increase the flexibility and dynamics of this region to cope with the complex environment of eukaryotic cells (35). The MD subsequently regulates the function of HSP90 by binding the γ-phosphate of NTD-specific ATP, thereby activating its ATPase activity (36). The CTD is mainly responsible for protein dimerization (37). In addition, the HSP90 family consists of four main members, including HSP90α, HSP90β, Gp96, and tumor necrosis factor receptor-related protein 1 (Trap1) (34). Different members are distributed in different cellular compartments. Furthermore, HSP90α is an inducible thermoprotein, whereas HSP90β is a constitutive thermoprotein, both of which are located in the cytoplasm. The main difference between HSP90α and HSP90β is the presence or absence of glutamine fragments. Gp96 and TRAP1 are localized in the endoplasmic reticulum and mitochondria, respectively.

#### 2.1.1 HSP90 and cancer progression

HSP90 acts as a molecular chaperone to maintain normal cellular physiological functions, stabilizes signaling proteins and enhances tumor cell tolerance to stress, thus promoting tumor progression. HSP90 can bind protein kinases and transcription factors that are associated with cellular signaling; moreover, it can amplify signals through a series of relay proteins, thereby affecting the growth process of tumor cells. P53 acts as a tumor suppressor that regulates cell growth suspension, senescence, and apoptosis (38). In carcinoma cells, HSP90 can effectively stabilize the structure of mutant p53 and significantly inhibit its degradation. When p53 is point mutated, its function is impaired, and the accumulation of missense p53 in cancer cells promotes growth and metastasis (39, 40). Specifically, mutant p53 proteins acquire new procarcinogenic functions that lead to the disengagement of cells from the normal apoptotic process, which correspondingly promotes the development of cancerous lesions. Notably, the use of 17-AAG (an HSP90 inhibitor) was able to induce the release of mutant p53 from the complex, thus resulting in effective ubiquitination and degradation of p53. In addition, increased HSP90 expression promotes the activation of multiple oncogenic protein kinases and increases the stability of proteins in signaling pathways such as the TGF-β, MAPK, AKT/PI3K, and WNT pathways; this effect maintains the growth signaling pathway and further promotes tumor growth (41).

### 2.2 HSP70

HSP70 (HSP72 or HSPA1) is the most important member of the heat shock protein family; it has a molecular weight of approximately 70 kDa and contains more than 20 different proteins with molecular weights of 68, 72, 73, 75, and 78 kDa. HSP70 consists of two highly conserved structural domains: a nucleotide binding site (NBD) at the N-terminal end and a substrate binding site (SBD) at the C-terminal end (42). The HSP70 family consists of at least eight homologous chaperone proteins that are mainly distributed in the nucleus and cytoplasm. The proteins of this family can be divided into three categories: stress-inducible HSP70, constitutive HSP70, and specific proteins localized in different organelles, such as HSP75 (mitochondria) and GRP78 (endoplasmic reticulum) (43, 44). Under physiological conditions, HSP70 is mainly located in the cytoplasm and functions by assisting in the folding of newly synthesized polypeptides, the assembly of multiprotein complexes, and the transport of proteins across the cell membrane. Under severe stress conditions, HSP70 levels increase rapidly in the nucleus, whereas only a small amount of HSP70 is present in the cytoplasm; moreover, when the cell recovery phase begins, HSP70 gradually disappears from the nucleus, whereas the cytoplasm still maintains a low level of HSP70 expression (45). In addition, HSP70 prevents protein aggregation, promotes refolding of misfolded proteins, and solubilizes aggregated proteins. It also acts synergistically to remove aberrant proteins and their aggregates via cellular degradation mechanisms (46).

#### 2.2.1 HSP70 inhibits apoptosis to promote tumor progression

HSP70 promotes the survival of tumor cells. After exposure of tumor cells to stressful environments, the synthesis of HSP70 in cancer cells is accelerated, and HSP70 accumulates in tumor cells. High levels of HSP70 in the cytoplasm protect cancer cells from apoptosis, promote tumor cell proliferation and migration, and aid in therapeutic resistance, thus leading to an aggressive tumor phenotype (47). HSP70, which is distributed on the lysosomal membrane of tumor cells, resists tumor cell death by mediating membrane stability, inhibiting lysosomal membrane permeability, and preventing the release of tissue proteases (48). Inducible HSP70 blocks endogenous and exogenous apoptotic pathways to varying degrees, thus increasing the survival time of tumor cells in response to stressful stimuli such as anticancer drugs. HSP70 can block caspase-dependent and caspase-nondependent apoptotic pathways (49). In the caspase-dependent apoptotic pathway, HSP70 inhibits c-Jun N-terminal kinase (JNK) activity by promoting the proteasomal degradation of apoptosis signal-regulated kinase 1 (ASK1) (50). JNK activity plays an important role in promoting mitochondrial cytochrome c release and inducing apoptotic pathways (51). Intriguingly, HSP70 promotes the activation of the MAPK pathway when tumor cells are exposed to cisplatin oncolytic drugs, thereby protecting cancer cells from cisplatin-induced apoptosis (52). In addition, HSP70 plays an important role in regulating the mitochondrial pathway. At the premitochondrial level, HSP70 regulates apoptosis by inhibiting or negatively regulating the activity of related kinases, as well as by affecting the transcription of Bcl-2 family apoptotic proteins (53). Moreover, there is a protein-protein interaction (PPI) between HSP70 and Bcl-2-interacting mediator (BIM), which plays a protective role in mitochondrial autophagy and apoptosis; additionally, the protection of the complex against mitochondrial autophagy and apoptosis can be disrupted via S1g-2 (a Bcl-2-Bim interferon) (54). At the mitochondrial level, HSP70 binds to the proapoptotic protein Bax, thus preventing it from translocating to the mitochondria and inhibiting the release of apoptotic molecules from the mitochondria. Moreover, HSP70 prevents the recruitment of apoptotic vesicles at the postmitochondrial level (55).

### 2.3 HSP60

HSP60, which is also known as HSPD1 or CPN60, is a stressed heat shock protein that is predominantly found in mitochondria (56). Initially, HSP60 was thought to be localized only in mitochondria; however, as research progressed, it was found to be equally present in the cytoplasm, nucleus, and blood circulation. The N-terminus of HSP60 has a mitochondrial localization sequence that contributes to its localization to mitochondria, whereas the C-terminus contains a series of G-repeat sequences (57). Notably, HSP60 usually forms an asymmetric complex with the cochaperone protein HSP10, whereby these proteins work together in the process of folding and in correcting the misfolding of proteins in the cytoplasm (58).

#### 2.3.1 Double-edged sword of HSP60 tumor progression

HSP60 regulates apoptosis via various interactions and messaging from inside and outside of the cell (59). HSP60 has dual roles in antagonizing apoptosis and promoting apoptosis, and the balance between the two effects is thought to be critical in the pathogenesis of cancer. Erbao et al. knocked down the expression of HSP60 via the lentivirus RNA interference technique (lenti-siRNA), and reported that when the cells were exposed to heat stress, the number of apoptotic cells increased with increasing heat stress. These findings demonstrate that HSP60 prevents apoptosis by stabilizing the inner and outer mitochondrial membranes (60). The NF-κB gene, which is a key regulator of cell survival, contains numerous antiapoptotic genes. Cytoplasmic HSP60 promotes the activation of the NF-κB survival pathway via direct interactions with cytoplasmic IKKα/β, which is the central activating enzyme of NF-κB (61). In addition, HSP60 promotes the translocation of BAX to mitochondria in the cytoplasm and its insertion into the outer mitochondrial membrane, where it forms BAX macropores; these micropores subsequently modulate the permeability of the outer mitochondrial membrane and induce apoptosis (62).

## 3 HSP-Ex: a TIME regulator

### 3.1 HSP90-Ex: an initiator of tumor microenvironment reprogramming

HSP90-Exs have been shown to function as cellular communicators in the tumor microenvironment (TME), which is largely dependent on their specific location on the membrane surfaces of exosomes (63). HSP90-Exs are involved in two mechanisms, including the activation of metalloproteinase-2 (MMP-2) and human epidermal growth factor receptor-2 (HER-2) and the activation of fibrinogen during tumor metastasis. The precise molecular mechanism by which HSP90-Exs activate MMP-2 is not clear; however, HSP70 is clearly an indispensable coinitiator (64). Notably, HSP90 directly interacts with HER-2 and activates HER-2 downstream molecular pathways to direct cytoskeletal rearrangement and enhance tumor cell invasive properties (65). In addition, it has been reported that breast cancer and glioblastoma cells secrete HSP90-Exs to promote the conversion of fibrinogen to the active form of fibrinolytic enzymes. Specifically, HSP90, protease tissue plasminogen activator (tPA) and membrane-associated protein II are a set of ternary complexes on the surfaces of HSP90-Ex membranes that aid in plasminogen activation (66, 67). The active protease form of fibrinolytic enzymes reprograms the local matrix of the TME and increases the probability of tumor cells in binding to migratory attachment sites. Interestingly, high levels of HSP90-Exs were detected in metastatic oral squamous cell carcinoma (OSCC.) However, the knockdown of HSP90 alone did not significantly reduce tumor metastasis. Both HSP90α and HSP90β must be knocked down via small interfering RNA (siRNA) to effectively inhibit tumor metastasis (68).

**Figure 1:**
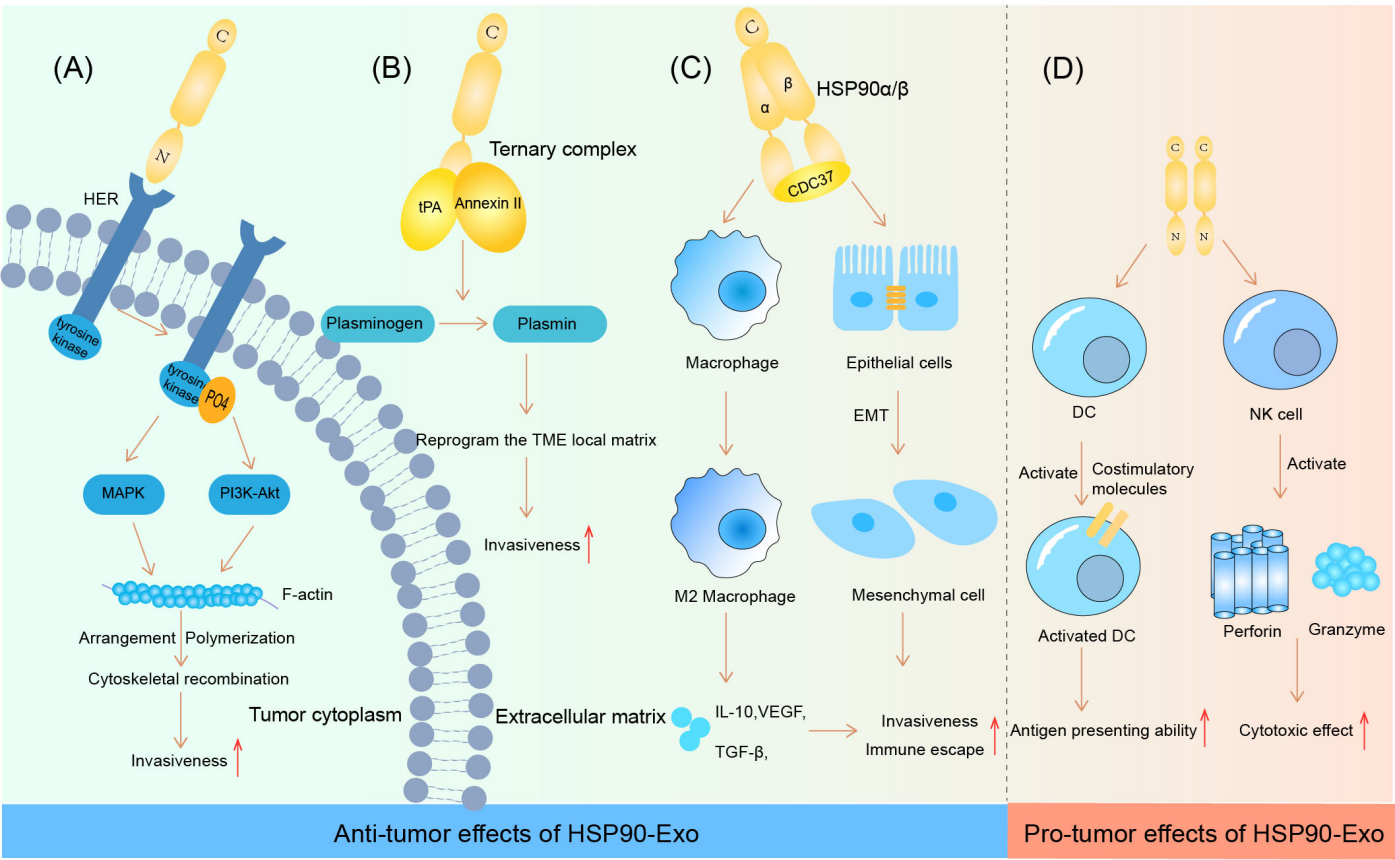
HSP90-Ex’s bilateral modulation in the tumor microenvironment. Pro-tumorigenic Actions: **(A)** HSP90 engagement with HER induces HER-2 tyrosine phosphorylation, activating the MAPK and PI3K-Akt cascades. This activation pattern influences F-actin dynamics, leading to cytoskeletal rearrangements essential for tumor cell invasion and migration. **(B)** The HSP90-Ex membrane from breast and glioblastoma cells features a trimeric complex that activates plasminogen, degrading fibrin in the ECM and facilitating tumor cell migration. **(C)** In oral cancer, a complex of HSP90α/β and CDC37 promotes EMT in epithelial cells, enhancing cancer cell migration, invasion, and tumorigenesis. HSP90β accumulation in TAMs induces an M2 polarization, supporting oncogenic progression. Anti-tumorigenic Actions: **(D)** HSP90-Ex interaction with DCs triggers NF-kB activation, enhancing DC functions and antigen presentation. Exosomes from hepatocellular carcinoma cells, enriched with HSPs, stimulate NK cell receptors upon drug treatment, activating NK cells to secrete cytotoxic molecules, thus bolstering anti-tumor responses.

Recent studies have shown that HSP90-Exs, which are abundant in the TME, can induce M2-type polarization of macrophages by mediating CD91 on the macrophage surface. This process promotes the progression of pancreatic ductal adenocarcinoma (PDAC), as M2-type macrophages play an important role in the construction of the immunosuppressive tumor microenvironment (69). Similarly, the depletion of HSP90α/β and cell division control-37 (CDC37) ternary complexes via siRNA in OSCC significantly reversed the outcome of tumor progression. This is due to the ability of the ternary complex to encourage the chemotactic M2-type polarization of macrophages, as well as the epithelial-mesenchymal transition (EMT) (70). In addition to the negative immune response triggered by the chemotaxis of M2-type macrophages, HSP90-Exs have also been shown to play a role in activating dendritic cells (DCs) to initiate an adaptive immune response. Studies have shown that HSP90-Exs are able to activate DCs via the nuclear factor kB (NF-κB) pathway, which promotes antigen-presenting ability and costimulatory molecule expression, thereby exerting antitumor immunity (71). In addition, the release of HSP90-Exs by human hepatocellular carcinoma cells has been shown to activate NK cell cytotoxicity, as well as granzyme B production, which is an important factor in the development of a positive tumor immune microenvironment (72). The specific effects of HSP90-Exs on tumor progression are summarized in **Figure 1**.

### 3.2 HSP70-Exs: multiple messengers involved in tumor progression

HSP70 cannot leave the cell via the traditional transport pathways of the endoplasmic reticulum and Golgi proteins because it does not have a homologous transmembrane sequence (73). However, HSP70 is able to separate from the cell via other pathways that are related to the environment in which the cell is located and the signaling pathways that are activated. One mechanism involves tumor cells secreting HSP70-carrying membranous vesicles via plasma membrane vesicles, which are usually 40–150 nm in diameter; these vesicles are also known as tumor-derived exosomes (TDEs), and they play a communication role in regulating the TIME (74). Notably, HSP72 on the surfaces of tumor-derived exosomes (TDEs) can produce IL-6 to trigger Stat3 activation in MDSCs in a TLR2/MyD88-dependent manner, which subsequently inhibits T-cell differentiation. However, exosomes from the 3T3 cell line (normal cell line) have no immunosuppressive function. Therefore, this mechanism is sufficient for HSP-Exs to have the specific ability of editing the TIME (75).

Membrane-bound HSP70 (mHSP70) released from tumor cells under the influence of interleukin-2 (IL-2) has been shown to activate natural killer (NK) cells (76). When NK cells are activated, they produce granzyme B, and mHSP70 facilitates the uptake of granzyme B by HSP70-positive tumor cells in a nonperforin-dependent manner. This process subsequently triggers apoptosis via a cysteine-dependent pathway. Intriguingly, researchers cocultured human NK cells with peptides that are present in the external environment of tumor cells (e.g., full-length heat shock protein 70 or a 14-amino acid peptide derived from the C-terminal structural domain of HSP70), which activated NK cells (CD57+/CD94+ NKs) and induced the migration of these cells to recognize and destroy the membranes of HSP70-positive tumor cells (77).

It is promising that exogenous heat shock protein 70 (eHSP70) can send early warning signals to the innate and adaptive immune systems and play a key role in the remodeling process of the TIME. eHSP70 interacts with CD14 and Toll-like receptors 2 and 4 (TLR2/4) to activate NF-κB and prompts antigen-presenting cells (APCs) to release proinflammatory cytokines such as nitric oxide (NO), IL-1β, IL-6, IFN-γ, and TNF-α (78–80). From another perspective, it is possible that HSP70/Bag-4 surface-positive TDEs released by human pancreatic and colorectal cancer cells trigger innate immune responses by activating NK cells (81). NK cells treated with full-length HSP70 and exposed to TKD peptides exhibited effects similar to those described in previous studies (82).

Notably, eHSP70 can trigger IFN-γ production by NK cells, and this induced effect is based on an interactive exchange between NK cells and DCs. This interaction occurs via the binding of the NK cell activation receptor NKG2D to the NKG2D ligand known as MHC type I chain-associated protein A (MICA) on the surface of DCs. Furthermore, eHSP70 has the ability to prompt DCs to express MICA (83). In addition, HSP70 can also induce DC maturation by increasing the expression of MHC class II molecules and other costimulatory molecules (such as CD86, CD83, and CD40). In this process, peptides 407–426 in the C-terminal structural domain of HSP70 are involved in cytokine production (84).

Following activation, mature CDs interact with CD8+ cytotoxic T lymphocytes (CTLs) to trigger and generate an adaptive immune response.

Tumor antigen-peptide complexes that bind to eHSP70 interact with receptors on the surfaces of APCs, including dendritic cells, macrophages, and monocytes, as well as endothelial and epithelial cells. These receptors include CD36, CD91, CD14, CD40, and TLR2/4, as well as scavenger receptors such as LOX-1, SR-A, SREC-1, and FEEL-1. Recently, these receptors have been identified as being possible binding sites for eHSP70 (85). HSP70 receptor-mediated endocytosis is facilitated by this interaction, which results in the interaction of the antigen with the major MHC class I molecules in the histocompatibility complex for cross-delivery, thereby triggering an antitumor immune response (86, 87).

Notably, the substrate-binding domain (SBD) of HSP70 plays a key role in the transport of antigens and their binding to MHC class I molecules (88). Other studies have shown that eHSP70, which does not bind to peptides, triggers a cytotoxic response in helper T-cells, thereby stimulating an adaptive immune response (89). eHSP70 not only transmits stimulatory immune signals as autocrine and paracrine signals in TDEs but also plays a role in tumors, the immune system, endothelial cells, and epithelial cells. These signals can stimulate the inhibitory mechanisms of the immune system, create an inflammatory microenvironment, trigger the activation of MMPs, and accelerate blood vessel formation, thereby accelerating the growth and spread of tumor cells.

**Figure 2:**
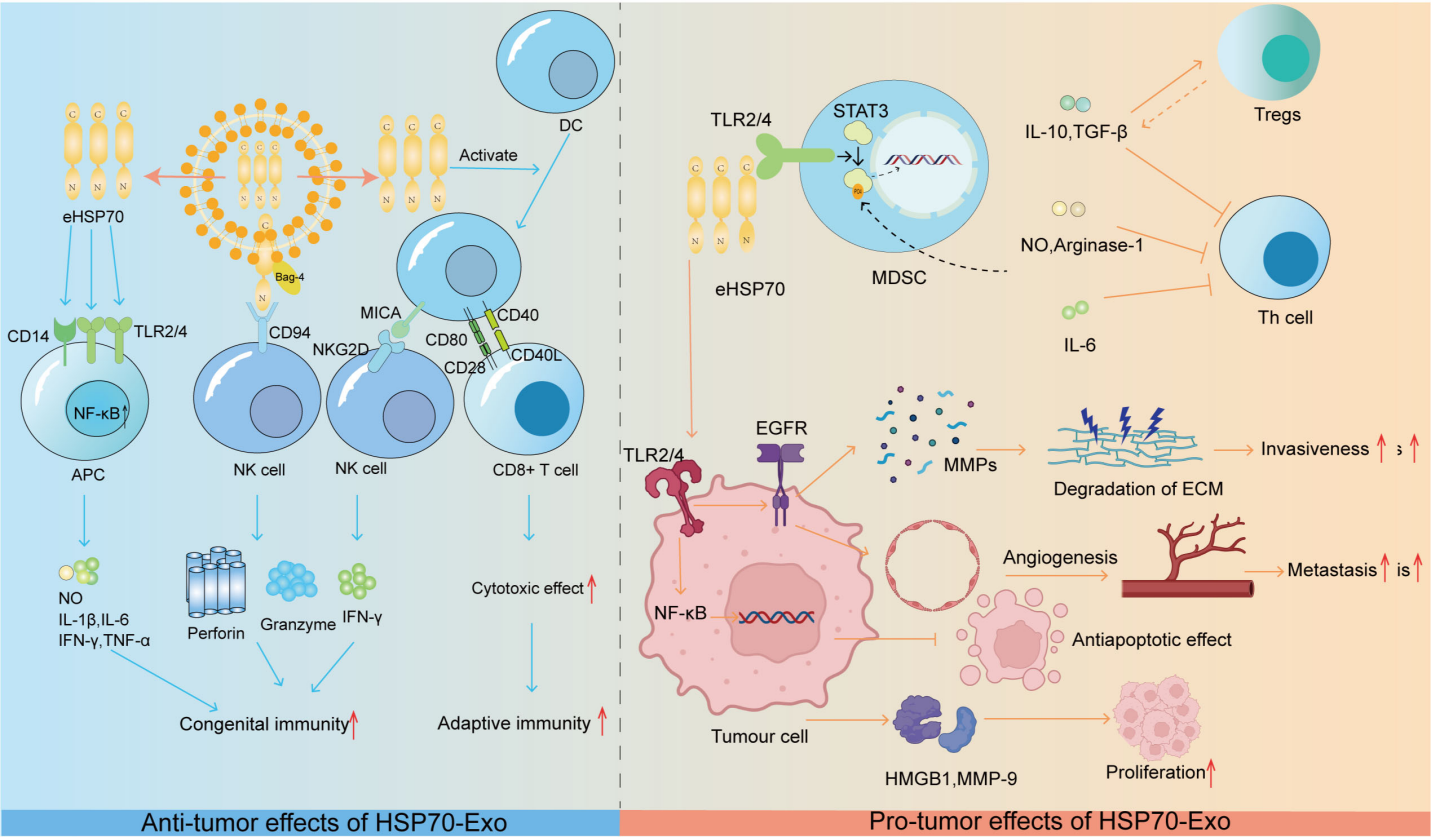
HSP70-Ex: orchestrating tumor progression through the immune microenvironment. Anti-tumorigenic Role: HSP70 exerts anti-tumorigenic effects by modulating innate and adaptive immune responses.eHSP70 from exosomes interacts with CD14 and TLR2/4 on APCs, activating the NF-kB pathway to stimulate the release of immunostimulatory molecules. The HSP70/Bag-4 complex activates NK cells, and eHSP70-induced MICA expression on DCs triggers NK cell cytotoxicity. Moreover, eHSP70 enhances DC co-stimulatory molecule expression, promoting CD8+ T-cell activation and anti-tumor cytotoxicity. Pro-tumorigenic Role: eHSP70 promotes tumorigenesis by modulating the immune microenvironment. It activates NF-kB and EGFR signaling in tumor cells, enhancing survival and promoting angiogenesis and MMP release. Additionally, it induces HMGB1 and MMP-9, fostering a pro-tumorigenic microenvironment. HSP70 in TDEs dose-dependently activates Stat3 in MDSCs, leading to the production of immunosuppressive factors that inhibit Th cell activation. IL-6, IL-10, and TGF-β further promote an immunosuppressive environment by stimulating MDSCs and Treg activation, respectively.

eHSP70 has been shown to have significant immunosuppressive effects on myeloid-derived suppressor cells (MDSCs) in experimental models in both mice and humans. From a macroscopic perspective, free eHSP70 has been shown to increase the immunosuppressive capacity of CD4+CD25+FoxP3+ regulatory T-cells (Tregs). This process results in a decrease in the levels of proinflammatory cytokines such as IFN-γ and TNF-α and an increase in the secretion of suppressive cytokines such as IL-10 and TGF-β (90). In addition, eHSP70 can also be regarded as a threat-associated molecular pattern (DAMP) that triggers increased cytokine production, which correspondingly creates an inflammatory microenvironment that contributes to tumor development (91). For example, HSP70 released from heat shock-treated A431 squamous cell carcinoma cells is able to activate the epidermal growth factor receptor (EGFR) and ERK1/2 signaling pathways via interaction with TLR2/4 (92). When considering the key role that epidermal growth factor receptors play in MMP activation and subsequent tumor invasion and metastasis, eHSP70 is likely involved in these biological processes (93). The NF-κB pathway triggered by eHSP70 binding to TLR2/4 on H22 hepatocellular carcinoma cells also has the potential to exhibit antiapoptotic properties that promote tumor growth (94). More specifically, eHSP70 accelerates tumor growth and progression by inducing the release of highly migratory group protein B1 (HMGB1) and the upregulation of MMP-9 in H22 hepatocellular carcinoma cells (95). Similarly, eHSP70 induces MMP-9 expression through the activation of NF-κB and activator protein-1 (AP-1) in human U937 monocytes (96). The specific effects of HSP90-Exs on tumor progression are summarized in **Figure 2**.

### 3.3 HSP60-Exs: initiator of antitumor immunity

Recent scientific studies have indicated that the exosome HSP60 has significant antitumor effects in a mouse B-cell lymphoma/leukemia tumor model (A20). In addition, exosomes isolated from heat shock lymphoma cells contain significantly more HSP60 than those produced by unstimulated cells (97). Increased HSP60-Ex levels result in an increased number of molecules associated with immunogenicity, including MHC class I, MHC class II, CD40, CD86, RANTES, and IL-1β. These HSP-Exs are able to more efficiently induce the phenotype and function of DCs, as well as CD8+ and CD4+ T-cells, to reach a mature state, which is essential for triggering tumor rejection. When investigated in a model of hepatocellular carcinoma and colorectal cancer, HSP60-Exs have the ability to stimulate cytotoxicity and granzyme B production in NK cells (72, 98).

HSP60-Exs are also affected by chemotherapeutic agents such as SAHA (serotonin linoleate), which is a histone deacetylase inhibitor (HDACi). SAHA may cause an increase in the secretion of HSP60-Exs, which is paralleled by a decrease in the cytoplasmic concentration of HSP60 (99). Nitrosation of HSP60 is caused by oxidative stress triggered by SAHA, and the molecular chaperones that are involved in nitrification are recognized in exosomes (100). Although nitrated HSP60-Exs can promote the immunogenicity-enhancing effect of SAHA on cancer cells, the molecular mechanisms that are involved in this effect remain to be elucidated (101). As observed with SAHA, the hepatocellular carcinoma cell line HepG2 demonstrated increased HSP60-Ex secretion after treatment with irinotecan HCl and carboplatin. These exosomes induce a stronger antitumor immune response than do those released from untreated cells (72).

## 4 Advances in the use of heat shock proteins as tumor vaccines

Tumor vaccination, which is often referred to as tumor immunization or tumor immunotherapy, is currently recognized as being one of the most promising research strategies for tumor treatment. Tumor vaccines usually consist of cell fragments containing tumor-specific antigens (TSAs), tumor-associated antigens (TAAs), or lysates of autologous and allogeneic tumor cells. By altering or eliminating their carcinogenicity and preserving their immunogenicity, these antigenic substances trigger an immune response against specific tumor-associated antigens (TAAs) upon entry into the organism, which enhances the immune system’s ability to resist and kill specific tumors (102, 103). Tumor vaccines can be divided into five main categories based on the different components within the vaccine, including cellular vaccines, synthetic peptide-protein vaccines, nucleic acid vaccines, viral vector vaccines and bacterial vector vaccines (104). These tumor vaccines are taken up by APCs via different components and are delivered to CD8+ T-cells and CD4+ T-cells via MHCI and MHCII molecules, thus causing the activation of effector T-cells. However, most tumors have low antigenicity, and the selection of a suitable TSA or TAA for vaccine design is not possible. However, HSPs can be preferentially detected on the surfaces of tumor cells and their surrounding microenvironment, which can correspondingly selectively target HSPs as a vehicle to generate tumor vaccines. Recent advances in heat shock protein peptide-based tumor vaccines are summarized in **Table 2**.

**Table 2:** Heat shock protein-peptide vaccines that have been clinically tested, the types of tumors that have been targeted, the results of trial progression, and the associated mechanisms of action.

Vaccine name	Tumor type	Experimental phase currently in progress	Current experimental results	Mechanism of action
NCT00293423. NCT02122822	Recurrent glioblastoma multiforme	Phase I and II clinical trials, single-arm trials, clinical trials	Safety and preliminary efficacy; primary diagnostic role; improvements in survival and prognosis	Antigen-presenting cells systemically deliver tumor antigenic peptides of HSP-96, HSP-96 antigenic peptides bind to CD91 receptors on dendritic cells, the HSPPC-96 complex is internalized, and cross-delivery of cleaved tumor peptides on MHC class I and MHC class II promotes a robust CD4+ and CD8+ T-cell immune response (105–107)
Peptide vaccine binding 96 kd chaperonin				
HSP105‐derived peptide vaccine	Esophageal cancer, colon cancer	Phase I clinical trial	Inducing immune effects and improving the prognosis of patients with tumors	CTLs are widely aggregated at the injection site, as well as in the peripheral blood and tumor tissue (108)
Autologous tumor-derived heat shock protein gp96 peptide complexes	Metastatic melanoma	Phase I and II clinical trials	Feasible and with no apparent toxicity	Increased recognition of autologous or HLA-A-matched allogeneic melanoma cells by PBMCs (peripheral blood mononuclear cells) (109, 110)
Autologous HSP purified from patient tumors vaccine	Diffuse large B-cell lymphoma	Animal model experiments	Safety and validity	Anti-tumor CD8+ and CD4+ T-cell crossover (111)
Vitespen (an autologous tumor-derived heat shock protein gp96 peptide complex vaccine)	Phase IV human melanoma :	Randomized phase III clinical trial	Safety and validity	Stimulating homologous T-cells (112, 113)
	Primary stage I/II/III renal cell carcinoma	A multicenter, open-label, randomized phase III trial	Low toxicity (concomitant adverse effects)	Enhanced toll-like receptor stimulants (113, 114)

### 4.1 Heat shock protein-peptide vaccines

Heat shock proteins possess four modes of application in tumor vaccines: 1) exogenous heat shock proteins act as classical exogenous antigens due to the inconsistency of their sequences with those of the host cells; 2) they are reactive because tolerance to self-heat shock proteins is broken or not yet fully established; 3) cross-reactivity between self-heat shock proteins and related proteins triggers an immune response to the latter protein type in the organism; and 4) HSP-antigen complexes induce an effective immune response (i.e., heat shock proteins act as carriers to facilitate the transport of antigenic substances). Currently, the fourth mode of cancer vaccine development is the most promising mode. The fourth mode is specific and capable of generating protective immunity against autologous (but not homologous) tumors. HSP-peptide complexes isolated from healthy individuals fail to effectively trigger tumor immune responses (115). From the perspective of tumor types, cancers have unique antigenic properties. Individual cancers can be vaccinated against their own tumors with specific vaccines; however, such vaccines are not effective against other types of cancers. Therefore, heat shock protein-peptide vaccines that are designed for specific cancers are only effective against the same type of cancer (116). After the vaccine enters into an organism, the organism begins to present the antigen. The binding of APC to the heat shock protein-peptide complex involves multiple receptors and is saturable (117). For example, CD91 (an LDL-associated protein) (118), LOX-1 (a low-density lipoprotein) (119) and Toll-like receptors and their cofactors CD14 and CD40 have been shown to be involved in the binding of heat shock protein-peptide complexes to APCs (120). HSP binding to these receptors promotes APC maturation (121). Although HSP is considered an “exogenous antigen”, it can be converted to the MHC class I pathway via the endogenous MHC class II pathway, which is a process known as “antigen cross-presentation” (86). A clinical study revealed that CD8+ T-cells are required for effective tumor rejection (122). Exogenous antigens are usually presented via MHC class II molecules; however, heat shock proteins are able to enter into the endogenous pathway and consequently be presented to MHC class I molecules (123). Numerous studies have shown that macrophages take up antigens via specific mechanisms. When a macrophage binds to an HSP complex, the HSP acts as a carrier to separate from the peptide complex and is processed and presented in the context of the macrophage’s own MHC class I molecules. Even if only 1% of the specific antigenic peptides are efficiently processed, approximately 107 specific antigenic peptides can still be obtained. These antigenic peptides are sufficient to stimulate T-cells, which results in strong stimulation of the immune system, thus resulting in the unusually high specificity and immunogenicity of the HSP peptide complexes (115). The binding of macrophages to a specific CD8+ T-cell epitope stimulates T-cell clones that are specific for that epitope (86). After vaccination with the HSP peptide vaccine, vaccination with the HSP peptide complex was shown to induce long-lasting memory T-cell immunity, as well as resistance to radiation, which represent characteristics of an idealized vaccine with long-term immune memory function (124).

### 4.2 HSP fusion vaccine

#### 4.2.1 HSP fusion cell vaccine

Cell fusion technology involves the process of fusing tumor cells with DCs. By fusing with tumor cells, DCs are able to obtain the assistance of HSPs to improve the efficiency of antigen cross-presentation. This process implements an antigen presentation mechanism that effectively introduces tumor antigens into DCs (125). It has been demonstrated that ovarian cancer cells or human breast cancer cells and DCs can be fused to rapidly isolate the HSP70 peptide complex (PC-F), in order to obtain an HSP fusion vaccine. This vaccine primarily targets the molecular chaperones of tumors and is prepared by extracting patient autologous tumor cells, which provides specificity and safety (126). The experimental data indicated that PC-F was involved in the Ag processing of DC-tumor fusion cells, whereas PC-F carried elevated levels of tumor antigenic peptides that enhanced T-cell responses to tumor cells. PC-F was able to induce T-cells to express higher levels of IFN-γ than HSP70-stimulated T-cells alone and reverse the immune tolerance of cancer cells. This implies that the fusion cell vaccine interrupts T-cell unresponsiveness to unmutated tumor antigens and results in increased levels of tumor cell death (125).

#### 4.2.2 HSP genetic fusion vaccines with tumor-specific antigen genes

HSP genes combined with tumor antigen genes can also function as antitumor agents. Studies of glioma treatment with an epitope fusion protein of HSP70 and NY-ESO-1 (a tumor immunotherapy cancer testis antigen) have shown significant enhancement of CTL-mediated cytotoxicity and *in vitro* targeting against NY-ESO-1-expressing tumors (127). Mucin 1 (Muc1) is a tumor-specific antigen that is overexpressed in several adenocarcinomas. Choi et al. designed a chimeric vaccine by fusing the human HSP70 gene with the Muc1 gene, which has a deletion of the C-terminal structural domain, after which they inoculated mice with this vaccine. It was observed that antigen-specific lymphocyte proliferation and cytotoxicity were effectively induced in mice that were vaccinated with the chimeric Muc1 vaccine (128). MAGE-3 is expressed in tumors of various histological types and is a member of the melanoma antigen (MAGE) gene family, which is capable of inducing antigen-specific immune responses *in vivo*, thus making it an ideal material for tumor vaccines. In previous studies, researchers attached the amino acids of MAGE-3 to the C-terminus of HSP70 and reported that the HSP70-MAGE-3 fusion protein triggered better cellular and humoral immune responses against MAGE-3-expressing mouse tumors than did the MAGE-3 protein *in vivo*. These findings suggest that the HSP70-MAGE-3 fusion protein produces efficient antitumor immunity against MAGE-3-expressing tumors (129).

### 4.3 HSP-Exs as cancer vaccines

HSP-Exs are membrane vesicles containing HSPs released by tumor cells. These exosomes are capable of carrying a variety of biologically active molecules, such as antigens, lipids, miRNAs and immunostimulatory factors. As ideal carriers in immunotherapy, exosomes have good biocompatibility. Exosomes function in a relatively gentle manner with cells, thus reducing adverse reactions to the body compared with traditional drug transport carriers (130). HSP-Exs are involved in the regulation of immune activity via multiple mechanisms, including 1) antigen presentation, 2) cytokine release, and 3) regulation of the TME. Based on the abovementioned properties, HSP-Exs can be used as ideal tumor vaccines. The production of the HSP-Ex vaccine is mainly elicited via heat shock treatment of tumor cells to collect and obtain exosomes in the culture medium, after which filtration is performed (such as fractional ultracentrifugation, polymer precipitation and membrane filtration) to efficiently purify the exosomes. The transfection of specific antigen genes into tumor cells via genetic engineering to obtain exosomes that are enriched with the target antigen is another methodological approach (131).

## 5 The potential of HSP-Exs as cancer vaccines and their clinical applications

In the immune system, exogenously acquired peptides undergo multiple pathways to reach specialized intracellular compartments; moreover, they are bound to MHC class I molecules and presented to APCs after selective and specific proteolytic processing (132). In tumor cells, HSPs act as chaperone proteins to maintain tumor cell survival in extreme environments. Furthermore, in terms of antitumor effects, HSPs can first be effectively internalized by antigen-presenting cells via receptor-mediated endocytosis. These internalized antigens can subsequently enter into different cellular compartments to assemble into new MHC molecules. Eventually, receptor-mediated internalization of chaperone proteins induces the phenotypic and functional maturation of APCs (133). The key to the success of tumor vaccines depends on the cross-presentation of tumor antigens by DCs. DCs can phagocytose and take up tumor cell-derived microparticles (T-MPs) (134). In phagosomes, HSPs can assist in the process by which T-MP-containing tumor antigens are degraded into tumor antigenic peptides.

**Figure 3:**
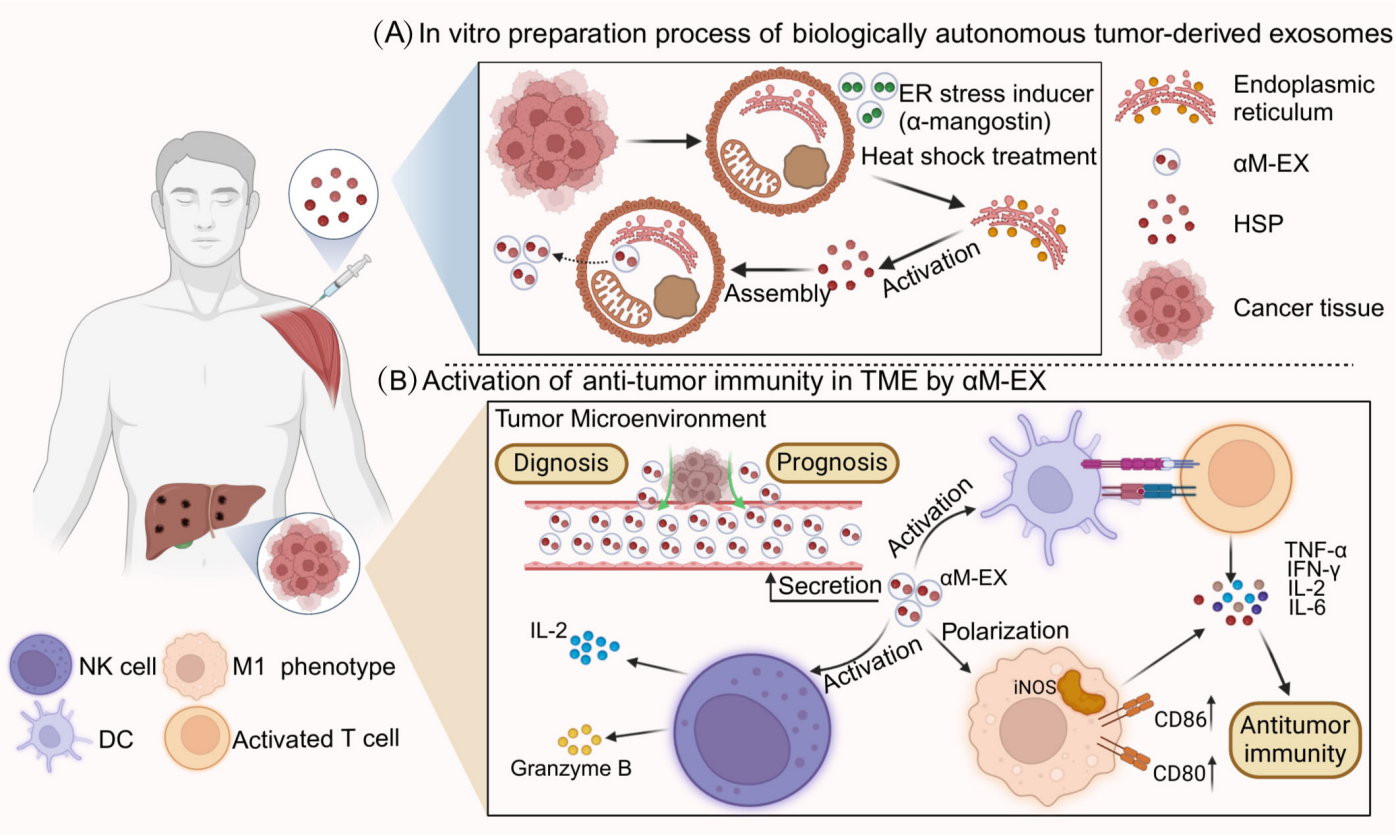
Preparation process and immune function of HSP-Ex. **(A)** part represents the heat stress treatment (e.g., α-mangostin) of tumor cells, which is processed by the endoplasmic reticulum and Golgi to generate a large number of tumor-derived exosomes (TDEs).TDEs are secreted into the tumor microenvironment and artificially collected through cellular cytokinesis. **(B)** part mainly demonstrates the clinical applications of HSP-Ex, in addition to its ability to act as a circulating marker, HSP-Ex is able to trigger strong anti-tumor immunity. For starters, HSP-Ex can assist in the maturation of antigen-presenting cells (APCs), which in turn activate T-cells to initiate active immunity. In addition, HSP-Ex specifically activates macrophages and NK cells. Macrophage polarization to an M1 phenotype and secretion of pro-inflammatory factors, and NK cell activation and secretion of granzyme B and pro-inflammatory factors can trigger anti-tumor immunity.

Tumor-derived exosomes (TEXs) possess a number of advantages with respect to their use as antitumor vaccines. First, the composition of the exosomes is clear and unambiguous. In addition, MHC class I and class II content can be measured, and these exosomes can be stored frozen for at least 6 months and are able to render antigens efficiently accessible to the APC, thus triggering and amplifying antigen-specific immune responses. TEXs constitute the full range of antigens of the tumor cells from which they originate; therefore, they should be able to more effectively immunize the host compared to immunization via a single antigen (135). HSPs have a strong adjuvant capacity and are highly enriched in both cancer cells and TEXs; moreover, HSPs can increase the immunogenicity of TEXs and enhance the efficacy of cancer vaccines (136). Therefore, the application of HSP-Exs in tumor vaccines is widely promising. The preparation process and immune function of HSP-Exs are summarized in **Figure 3**.

### 5.1 Advantages of the HSP-Ex cancer vaccine for immunotherapy

Compared with other biomimetic cancer nanovaccines, the HSP-Ex vaccine contains a broad spectrum of antigens, thus making it more difficult for tumor cells to evade immune surveillance (137). Exosomes have many advantages as antitumor vaccines: 1) the composition of exosomes can be determined, and MHC class I and class II contents can be measured; 2) they can be cryopreserved for at least 6 months; and 3) they can efficiently present antigens to APCs to initiate and amplify antigen-specific immune responses (15). Many studies have demonstrated that HSP-Exs have a promising future as tumor vaccines, and several HSP-Ex immune vaccines have been developed for different tumors.

CD4+ T-cells play a major role in preventing tumorigenesis, whereas CD8+ T-cells are the main antitumor cells. HSP-Exs can induce a significant increase in antitumor immune responses in preventative and therapeutic lymphoma experiments *in vivo* (138). For example, Florencia et al. targeted exosomes isolated from the ascites of very aggressive murine T-cell lymphomas. These exosomes were experimentally observed to be enriched in HSP70 and HSP90, and these exosome vesicles were then inoculated into naive homozygous mice. The results of this study revealed that these exosomes induced humoral and cellular immune responses (especially specific immune responses) with concomitant induction of immune memory (139). In addition, HSP70-enriched exosomes have been shown to activate the immune system and suppress tumor cells to a greater degree than other exosomes in fibrosarcoma (140). These findings demonstrate that HSP-Ex inoculation can stimulate specific immune responses in the body. Therefore, the use of HSP-Exs as a tumor vaccine can activate the body’s immune system and reprogram the TIME to inhibit tumor progression.

Thermotherapy is now becoming known as a new therapeutic trend due to the limitations of conventional treatments in patients with breast cancer. Heat-stressed tumor cells release exosomes (HS-TEXs) containing carcinoembryonic antigen, HSP70 and MCH I to activate DCs (141). Based on these findings, Sen, Kacoli et al. investigated the secretion of HSP70-Exs in breast cells after thermotherapy and reported that exosomes secreted by breast cancer cells increased the HSP70 content under the stress-inducing conditions of thermotherapy (142). Intriguingly, HSP70 or gp96 stimulates macrophages to induce nitric oxide synthase (iNOS) expression, thus resulting in M1 macrophage polarization (79). Based on the abovementioned results, thermotherapy combined with HSP70-Ex vaccine therapy may have a more comprehensive therapeutic effect on breast tumors. In addition to endogenous HSP-Exs, there is a growing trend toward mimicking the HSP structure *in vitro* to activate the body’s immunity. In this manner, antigens can be efficiently captured and transported to DCs, thus activating antitumor immunity (143). All of the abovementioned studies demonstrated that tumor-derived HSP-Exs have a good ability to promote the tissue immune response. In addition, the feasibility of the use of HSP-Exs as tumor vaccines has been demonstrated in the context of biomaterial synthesis.

Currently, *in vitro* modifications of exosomes for cancer treatment are also receiving widespread attention. Modifications of exosomes at the nanometer level can achieve targeting effects, which provides a basis for the synthesis of HSP-Ex vaccines. Using glioblastoma-derived exosomes as a drug delivery system, researchers have synthesized biologically safe exosomes known as “Exo&TDPs”, which can cross the blood-brain barrier and deliver the chemotherapeutic drugs temozolomide (TMZ) and adriamycin (DOX) to glioblastoma tissues. The activation of antitumor immunity kills tumor cells (144). The abovementioned studies demonstrated the high degree of plasticity of tumor-derived exosomes and the possibility of encapsulating HSPs in exosomes for the production of HSP-Ex-based tumor vaccines (145, 146). The development of biologically self-assembled tumor cell-derived cancer nanovaccines offers a novel avenue for exploring ideal cancer immunotherapy. In another study, researchers introduced the ER stress inducer α-mangostin (αM) into melanoma cells via poly(D,L-propylidene-coglycolide) nanoparticles. A biologically self-assembled tumor cell-derived cancer nanovaccine (αM-EX) was subsequently harvested based on the biological process of extracellular throughput of the nanoparticles by the tumor cells. Via ELISA, researchers reported that the expression of tumor antigens and HSP70s was increased in αM-EX and that HSP70 significantly reduced the expression of tumor metastasis-associated proteins. HSP70- rich αM-EX have a strong ability to inhibit tumor growth, which is primarily achieved in the following ways. αM-EX possess a considerable quantity of 70 kDa heat shock proteins (Hsp70s), which are induced by ER stress. These proteins serve not only as endogenous adjuvants but also enhance LN targeting and DC internalization. After injection, αM-EX effectively migrates to LNs and is rapidly endocytosed by DCs, synchronously delivering tumor antigens and adjuvants to DCs, and then strongly inducing an anti-tumor immune response and establishing long-term immune memory. Additionally, the natural targeting of APCs by HSP70s is able to activate the endocytosis of bone marrow-derived dendritic cells (BMDCs) via the presentation of potential antigens and adjuvants (147). Exosome nanostructures enriched with HSPs can enhance the immune system’s response to tumors, whereas the adjuvant HSP70s contained in this material are an endogenous component of the cells, thus eliminating the risk of toxicity from exogenous adjuvants. Similarly, in the synthesis of vaccines for the treatment of established HPV lesions and malignant tumors, researchers have utilized various HSP27 proteins, including recombinant HSP27-E7 protein, tumor cell lysate (TCL-HSP27-E7), and HSP27-E7-Ex. The results showed that all three compounds induced an immune response in the body. Crucially, the presence of higher levels of granzyme B (which is secreted by cytotoxic lymphocytes) in the HSP27-E7-Ex regimen was able to target mitochondrial respiratory chain complex I, thus leading to ROS-dependent cell death (148). Thus, the HSP27-E7-Ex regimen has better tumor killing ability, in addition to a greater safety profile, and is considered to be the most promising strategy for HPV vaccination (149).

The abovementioned studies illustrate that the development and application of HSP-Exs as tumor vaccines are promising. Notably, despite the ability of HSP-Exs to activate antitumor immune responses, their use for tumor therapy is still not completely reliable. This is due to the fact that, in addition to this function, HSP-Exs have a series of contradictory functions, such as recruiting immunosuppressive responses, providing an inflammatory microenvironment, and activating MMPs and angiogenesis to promote tumor cell progression and metastasis. The ability of HSP-Exs to promote apoptosis and tumorigenesis has been demonstrated in different experimental models. Therefore, in the process of using HSP-Exs as tools for actual tumor therapy, a strategy should be selected according to the specific type of cancer, and the elucidation of the immunostimulatory mechanism of exosomes, which ensures that HSP-Exs can be carriers of antigens and adjuvants for cancer vaccines, is crucial.

### 5.2 Clinical applications of HSP-Exs

Clinical studies have detected high levels of HSP90-Exs in bodily fluid samples from patients with melanoma (150), papillary thyroid tumors (151), and rectal cancer (152). A cohort analysis was performed on more than 1,500 participants recruited from hospitals, which included patients with different types of cancer (including liver, lung, breast, colorectal, gastric, pancreatic, and esophageal cancers and lymphoma), as well as healthy individuals. The results showed that the detection of HSP90-Ex levels in plasma could identify 80% of cancer patients, even in the early stages of cancer development. HSP90-Ex is particularly important for cancer diagnosis in patients with high expression of ADAM10, which is a surface protein encoded by the chromosome 15 gene (153). Clinical studies have demonstrated that HSP-Exs can be used as circulating markers to predict the probability of cancer in patients.

In contrast, HSP70-Exs are also clinically used for disease diagnosis; for example, HSP70-Exs can be used for the early diagnosis of cancer. Related studies have revealed that noncancer cells (normal cells) release exosomes that do not express HSP70; however, tumor cells release high levels of exosomes and express HSP70 on their membrane surfaces (154). This study analyzed HSP70-Ex in combination with currently used biomarkers, including carcinoembryonic antigen (CEA) and carcinoembryonic antigen 19-9 (CA19-9), by measuring HSP70-Ex levels in a group of patients with lung cancer and in a group of healthy patients; the results suggested that HSP70-Ex can be used for the initial diagnosis of early-stage lung cancer (155). HSP70-Ex is a superior biomarker for predicting tumor metastasis compared with circulating tumor cells (CTCs), which represent a current clinical diagnostic and detection indicator for predicting cancer metastasis (156). This benefit is due to the availability of exosomes with a uniform systemic distribution, a wide detection range and strong protection (157). The presence of HSP70-Exos may additionally serve as a predictor of the response to treatment. During the immunophenotyping of peripheral blood lymphocytes via multiparametric flow cytometry, HSP-Ex levels were found to be sequentially elevated with increasing tumor stage and metastasis. Notably, HSP70-Ex was shown to be persistently low when the patient’s condition was stabilized. In addition, studies have demonstrated that HSP70-Exs are associated with prognostic survival in a variety of cancers, including lung cancer, head and neck squamous cell carcinoma, gastric cancer, breast cancer, and glioblastoma (158–162). HSP60-Ex homeostasis is a key factor for the pathogenesis of cancer. Increased protein levels of HSP60 have been detected in specimens of solid tumors such as breast and colon cancers; therefore, HSP60-Ex has great potential as being an indicator of cancer diagnosis and prognosis (163).

## 6 Summary and outlook

HSPs are widely recognized as being crucial factors in the development and progression of tumors. With advancements in research, HSP-Exs have emerged as significant entities in oncology. These nanosized vesicles, which encapsulate HSPs, play critical roles in tumor growth, metastasis, and intercellular communication, thereby providing new avenues in cancer therapy. This study examined various types of HSP-Exs and their effects on tumor progression, as well as the changes that they induce in the tumor immune microenvironment. Furthermore, it addresses the development of vaccines and their clinical applications that are based on the mechanisms associated with HSP-Exs.

Currently, HSP-based tumor vaccines are classified into three categories: HSP-peptide vaccines, HSP vaccines, and HSP-Ex vaccines. This article examines the advantages and potential of HSP-Ex vaccines in clinical applications, as well as their current limitations. Nanometer-scale HSP-Exs that are derived from cellular tissues have excellent biocompatibility and low toxicity. Consequently, the use of HSP-Exs for vaccine development demonstrates significant promise. Ongoing research highlights the considerable potential of HSP-Exs as tumor vaccines.

However, their application in tumor therapy remains uncertain. HSP-Exs can function as a double-edged sword. In the investigated experimental models, they have been shown to both promote tumor apoptosis and facilitate tumor development, thus revealing a series of contradictory relationships. Therefore, the elucidation of the immune mechanisms associated with exosomes and the targeting of specific tumor types are essential factors for developing effective therapeutic strategies. Although research indicates that HSP-Exs affect tumor growth, metastasis, and dissemination through various mechanisms, additional studies are necessary to elucidate the specific receptors, pathways, and expression patterns of the involved genetic material. Furthermore, within tumor treatment protocols involving HSP-Exs, further experimental investigations on the dosage of injected drugs, their effects on treatment outcomes, and potential adverse reactions in patients are essential. This research is critical for enhancing clinical translation and optimizing treatment strategies.

## References

[1] Qi J Li M Wang L Hu Y Liu W Long Z. National and subnational trends in cancer burden in China, 2005-20: an analysis of national mortality surveillance data. Lancet Public Health. (2023) 8:e943–e55. doi: 10.1016/S2468-2667(23)00211-6 38000889

[2] Siegel RL Giaquinto AN Jemal A. Cancer statistics, 2024. CA Cancer J Clin. (2024) 74:12–49. doi: 10.3322/caac.21820 38230766

[3] Wu J Liu T Rios Z Mei Q Lin X Cao S. Heat shock proteins and cancer. Trends Pharmacol Sci. (2017) 38:226–56. doi: 10.1016/j.tips.2016.11.009 28012700

[4] Lianos GD Alexiou GA Mangano A Mangano A Rausei S Boni L. The role of heat shock proteins in cancer. Cancer Lett. (2015) 360:114–8. doi: 10.1016/j.canlet.2015.02.026 25721081

[5] Zuo WF Pang Q Zhu X Yang QQ Zhao Q He G. Heat shock proteins as hallmarks of cancer: insights from molecular mechanisms to therapeutic strategies. J Hematol Oncol. (2024) 17:81. doi: 10.1186/s13045-024-01601-1 39232809 PMC11375894

[6] Yin L Yang Y Zhu W Xian Y Han Z Huang H. Heat shock protein 90 triggers multi-drug resistance of ovarian cancer via AKT/GSK3β/β-catenin signaling. Front Oncol. (2021) 11:620907. doi: 10.3389/fonc.2021.620907 33738259 PMC7960917

[7] Lee HJ Min HY Yong YS Ann J Nguyen CT La MT. A novel C-terminal heat shock protein 90 inhibitor that overcomes STAT3-Wnt-β-catenin signaling-mediated drug resistance and adverse effects. Theranostics. (2022) 12:105–25. doi: 10.7150/thno.63788 PMC8690924 34987637

[8] Huang Y Peng C Tang J Wang S Yang F Wang Q. The expression of heat shock protein A12B (HSPA12B) in non-Hodgkin’s lymphomas. Ann Transl Med. (2021) 9:1462. doi: 10.21037/atm-21-4185 34734014 PMC8506729

[9] Asgary R. Cancer care and treatment during homelessness. Lancet Oncol. (2024) 25:e84–90. doi: 10.1016/S1470-2045(23)00567-3 38301706

[10] Rui R Zhou L He S. Cancer immunotherapies: advances and bottlenecks. Front Immunol. (2023) 14. doi: 10.3389/fimmu.2023.1212476 PMC10484345 37691932

[11] Schiller JT Kreimer AR. An HPV vaccine from India: broadening possibilities for cervical cancer control. Lancet Oncol. (2023) 24:1288–9. doi: 10.1016/S1470-2045(23)00535-1 37949087

[12] Litwin MS Tan HJ. The diagnosis and treatment of prostate cancer: A review. Jama. (2017) 317:2532–42. doi: 10.1001/jama.2017.7248 28655021

[13] Su J Brunner L Ates Oz E Sacherl J Frank G Kerth HA. Activation of CD4 T-cells during prime immunization determines the success of a therapeutic hepatitis B vaccine in HBV-carrier mouse models. J Hepatol. (2023) 78:717–30. doi: 10.1016/j.jhep.2022.12.013 36634821

[14] Lahiri A Maji A Potdar PD Singh N Parikh P Bisht B. Lung cancer immunotherapy: progress, pitfalls, and promises. Mol Cancer. (2023) 22:40. doi: 10.1186/s12943-023-01740-y 36810079 PMC9942077

[15] Regimbeau M Abrey J Vautrot V Causse S Gobbo J Garrido C. Heat shock proteins and exosomes in cancer theranostics. Semin Cancer Biol. (2022) 86:46–57. doi: 10.1016/j.semcancer.2021.07.014 34343652

[16] Xie S Wang X Gan S Tang X Kang X Zhu S. The mitochondrial chaperone TRAP1 as a candidate target of oncotherapy. Front Oncol. (2020) 10:585047. doi: 10.3389/fonc.2020.585047 33575209 PMC7870996

[17] Duarte BDP Bonatto D. The heat shock protein 47 as a potential biomarker and a therapeutic agent in cancer research. J Cancer Res Clin Oncol. (2018) 144:2319–28. doi: 10.1007/s00432-018-2739-9 PMC11813397 30128672

[18] Duan X Iwanowycz S Ngoi S Hill M Zhao Q Liu B. Molecular chaperone GRP94/GP96 in cancers: oncogenesis and therapeutic target. Front Oncol. (2021) 11:629846. doi: 10.3389/fonc.2021.629846 33898309 PMC8062746

[19] Yang S Xiao H Cao L. Recent advances in heat shock proteins in cancer diagnosis, prognosis, metabolism and treatment. BioMed Pharmacother. (2021) 142:112074. doi: 10.1016/j.biopha.2021.112074 34426258

[20] Proia DA Kaufmann GF. Targeting heat-shock protein 90 (HSP90) as a complementary strategy to immune checkpoint blockade for cancer therapy. Cancer Immunol Res. (2015) 3:583–9. doi: 10.1158/2326-6066.CIR-15-0057 25948551

[21] Zhang M Bi X. Heat shock proteins and breast cancer. Int J Mol Sci. (2024) 25(2):876. doi: 10.3390/ijms25020876 38255948 PMC10815085

[22] Nagaraju GP Long TE Park W Landry JC Taliaferro-Smith L Farris AB. Heat shock protein 90 promotes epithelial to mesenchymal transition, invasion, and migration in colorectal cancer. Mol Carcinog. (2015) 54:1147–58. doi: 10.1002/mc.v54.10 24861206

[23] Lettini G Pietrafesa M Lepore S Maddalena F Crispo F Sgambato A. Heat shock proteins in thyroid Malignancies: Potential therapeutic targets for poorly-differentiated and anaplastic tumours? Mol Cell Endocrinol. (2020) 502:110676. doi: 10.1016/j.mce.2019.110676 31812782

[24] Rao R Nalluri S Fiskus W Balusu R Joshi A Mudunuru U. Heat shock protein 90 inhibition depletes TrkA levels and signaling in human acute leukemia cells. Mol Cancer Ther. (2010) 9:2232–42. doi: 10.1158/1535-7163.MCT-10-0336 PMC3008427 20663926

[25] Hoter A Naim HY. Heat shock proteins and ovarian cancer: important roles and therapeutic opportunities. Cancers (Basel). (2019) 11(9):1389. doi: 10.3390/cancers11091389 31540420 PMC6769485

[26] Ando K Oki E Zhao Y Ikawa-Yoshida A Kitao H Saeki H. Mortalin is a prognostic factor of gastric cancer with normal p53 function. Gastric Cancer. (2014) 17:255–62. doi: 10.1007/s10120-013-0279-1 23828548

[27] Moon A Bacchini P Bertoni F Olvi LG Santini-Araujo E Kim YW. Expression of heat shock proteins in osteosarcomas. Pathology. (2010) 42:421–5. doi: 10.3109/00313025.2010.493866 20632817

[28] Trieb K Lang S Kotz R. Heat-shock protein 72 in human osteosarcoma: T-lymphocyte reactivity and cytotoxicity. Pediatr Hematol Oncol. (2000) 17:355–64. doi: 10.1080/08880010050034283 10914045

[29] El-Meghawry El-Kenawy A El-Kott AF Hasan MS. Heat shock protein expression independently predicts survival outcome in schistosomiasis-associated urinary bladder cancer. Int J Biol Markers. (2008) 23:214–8. doi: 10.5301/JBM.2009.3728 19199268

[30] Behnsawy HM Miyake H Kusuda Y Fujisawa M. Small interfering RNA targeting heat shock protein 70 enhances chemosensitivity in human bladder cancer cells. Urol Oncol. (2013) 31:843–8. doi: 10.1016/j.urolonc.2011.07.007 21889367

[31] Teng R Liu Z Tang H Zhang W Chen Y Xu R. HSP60 silencing promotes Warburg-like phenotypes and switches the mitochondrial function from ATP production to biosynthesis in ccRCC cells. Redox Biol. (2019) 24:101218. doi: 10.1016/j.redox.2019.101218 31112866 PMC6526248

[32] Xu X Wang W Shao W Yin W Chen H Qiu Y. Heat shock protein-60 expression was significantly correlated with the prognosis of lung adenocarcinoma. J Surg Oncol. (2011) 104:598–603. doi: 10.1002/jso.v104.6 21671464

[33] Csermely P Schnaider T Soti C Prohászka Z Nardai G. The 90-kDa molecular chaperone family: structure, function, and clinical applications. A comprehensive review. Pharmacol Ther. (1998) 79:129–68. doi: 10.1016/S0163-7258(98)00013-8 9749880

[34] Hoter A El-Sabban ME Naim HY. The HSP90 family: structure, regulation, function, and implications in health and disease. Int J Mol Sci. (2018) 19(9):2560. doi: 10.3390/ijms19092560 30158430 PMC6164434

[35] Tsutsumi S Mollapour M Prodromou C Lee CT Panaretou B Yoshida S. Charged linker sequence modulates eukaryotic heat shock protein 90 (Hsp90) chaperone activity. Proc Natl Acad Sci U S A. (2012) 109:2937–42. doi: 10.1073/pnas.1114414109 PMC3287002 22315411

[36] Meyer P Prodromou C Hu B Vaughan C Roe SM Panaretou B. Structural and functional analysis of the middle segment of hsp90: implications for ATP hydrolysis and client protein and cochaperone interactions. Mol Cell. (2003) 11:647–58. doi: 10.1016/S1097-2765(03)00065-0 12667448

[37] Huai Q Wang H Liu Y Kim HY Toft D Ke H. Structures of the N-terminal and middle domains of E. coli Hsp90 and conformation changes upon ADP binding. Structure. (2005) 13:579–90. doi: 10.1016/j.str.2004.12.018 15837196

[38] Li D Marchenko ND Schulz R Fischer V Velasco-Hernandez T Talos F. Functional inactivation of endogenous MDM2 and CHIP by HSP90 causes aberrant stabilization of mutant p53 in human cancer cells. Mol Cancer Res. (2011) 9:577–88. doi: 10.1158/1541-7786.MCR-10-0534 PMC3097033 21478269

[39] Wiech M Olszewski MB Tracz-Gaszewska Z Wawrzynow B Zylicz M Zylicz A. Molecular mechanism of mutant p53 stabilization: the role of HSP70 and MDM2. PloS One. (2012) 7:e51426. doi: 10.1371/journal.pone.0051426 23251530 PMC3520893

[40] Oren M. Decision making by p53: life, death and cancer. Cell Death Differ. (2003) 10:431–42. doi: 10.1038/sj.cdd.4401183 12719720

[41] Kryeziu K Bruun J Guren TK Sveen A Lothe RA. Combination therapies with HSP90 inhibitors against colorectal cancer. Biochim Biophys Acta Rev Cancer. (2019) 1871:240–7. doi: 10.1016/j.bbcan.2019.01.002 30708039

[42] Zhang P Leu JI Murphy ME George DL Marmorstein R. Crystal structure of the stress-inducible human heat shock protein 70 substrate-binding domain in complex with peptide substrate. PloS One. (2014) 9:e103518. doi: 10.1371/journal.pone.0103518 25058147 PMC4110032

[43] Schmitt E Gehrmann M Brunet M Multhoff G Garrido C. Intracellular and extracellular functions of heat shock proteins: repercussions in cancer therapy. J Leukoc Biol. (2007) 81:15–27. doi: 10.1189/jlb.0306167 16931602

[44] Daugaard M Rohde M Jäättelä M. The heat shock protein 70 family: Highly homologous proteins with overlapping and distinct functions. FEBS Lett. (2007) 581:3702–10. doi: 10.1016/j.febslet.2007.05.039 17544402

[45] Munro S Pelham HR. An Hsp70-like protein in the ER: identity with the 78 kd glucose-regulated protein and immunoglobulin heavy chain binding protein. Cell. (1986) 46:291–300. doi: 10.1016/0092-8674(86)90746-4 3087629

[46] Rosenzweig R Nillegoda NB Mayer MP Bukau B. The Hsp70 chaperone network. Nat Rev Mol Cell Biol. (2019) 20:665–80. doi: 10.1038/s41580-019-0133-3 31253954

[47] Shevtsov M Huile G Multhoff G. Membrane heat shock protein 70: a theranostic target for cancer therapy. Philos Trans R Soc Lond B Biol Sci. (2018) 373(1738):20160526. doi: 10.1098/rstb.2016.0526 29203711 PMC5717526

[48] Nylandsted J Gyrd-Hansen M Danielewicz A Fehrenbacher N Lademann U Høyer-Hansen M. Heat shock protein 70 promotes cell survival by inhibiting lysosomal membrane permeabilization. J Exp Med. (2004) 200:425–35. doi: 10.1084/jem.20040531 PMC2211935 15314073

[49] Creagh EM Carmody RJ Cotter TG. Heat shock protein 70 inhibits caspase-dependent and -independent apoptosis in Jurkat T-cells. Exp Cell Res. (2000) 257:58–66. doi: 10.1006/excr.2000.4856 10854054

[50] Gao Y Han C Huang H Xin Y Xu Y Luo L. Heat shock protein 70 together with its co-chaperone CHIP inhibits TNF-alpha induced apoptosis by promoting proteasomal degradation of apoptosis signal-regulating kinase1. Apoptosis. (2010) 15:822–33. doi: 10.1007/s10495-010-0495-7 20349136

[51] Tournier C Hess P Yang DD Xu J Turner TK Nimnual A. Requirement of JNK for stress-induced activation of the cytochrome c-mediated death pathway. Science. (2000) 288:870–4. doi: 10.1126/science.288.5467.870 10797012

[52] Sheng L Tang T Liu Y Ma Y Wang Z Tao H. Inducible HSP70 antagonizes cisplatin−induced cell apoptosis through inhibition of the MAPK signaling pathway in HGC−27 cells. Int J Mol Med. (2018) 42:2089–97. doi: 10.3892/ijmm.2018.3789 PMC6108861 30066840

[53] Nylandsted J Rohde M Brand K Bastholm L Elling F Jäättelä M. Selective depletion of heat shock protein 70 (Hsp70) activates a tumor-specific death program that is independent of caspases and bypasses Bcl-2. Proc Natl Acad Sci U S A. (2000) 97:7871–6. doi: 10.1073/pnas.97.14.7871 PMC16637 10884417

[54] Song T Yin F Wang Z Zhang H Liu P Guo Y. Hsp70-Bim interaction facilitates mitophagy by recruiting parkin and TOMM20 into a complex. Cell Mol Biol Lett. (2023) 28:46. doi: 10.1186/s11658-023-00458-5 37237369 PMC10223935

[55] Wang X Chen M Zhou J Zhang X. HSP27, 70 and 90, anti-apoptotic proteins, in clinical cancer therapy (Review). Int J Oncol. (2014) 45:18–30. doi: 10.3892/ijo.2014.2399 24789222

[56] Ishida R Okamoto T Motojima F Kubota H Takahashi H Tanabe M. Physicochemical properties of the mammalian molecular chaperone HSP60. Int J Mol Sci. (2018) 19(2):489. doi: 10.3390/ijms19020489 29415503 PMC5855711

[57] Hansen JJ Bross P Westergaard M Nielsen M Eiberg H Børglum AD. Genomic structure of the human mitochondrial chaperonin genes: HSP60 and HSP10 are localised head to head on chromosome 2 separated by a bidirectional promoter. Hum Genet. (2003) 112:436. doi: 10.1007/s00439-003-0927-3 12483302

[58] Okamoto T Yamamoto H Kudo I Matsumoto K Odaka M Grave E. HSP60 possesses a GTPase activity and mediates protein folding with HSP10. Sci Rep. (2017) 7:16931. doi: 10.1038/s41598-017-17167-7 29208924 PMC5717063

[59] Chandra D Choy G Tang DG. Cytosolic accumulation of HSP60 during apoptosis with or without apparent mitochondrial release: evidence that its pro-apoptotic or pro-survival functions involve differential interactions with caspase-3. J Biol Chem. (2007) 282:31289–301. doi: 10.1074/jbc.M702777200 17823127

[60] Kim YE Hipp MS Bracher A Hayer-Hartl M Hartl FU. Molecular chaperone functions in protein folding and proteostasis. Annu Rev Biochem. (2013) 82:323–55. doi: 10.1146/annurev-biochem-060208-092442 23746257

[61] Chun JN Choi B Lee KW Lee DJ Kang DH Lee JY. Cytosolic Hsp60 is involved in the NF-kappaB-dependent survival of cancer cells via IKK regulation. PloS One. (2010) 5:e9422. doi: 10.1371/journal.pone.0009422 20351780 PMC2843631

[62] Song E Tang S Xu J Yin B Bao E Hartung J. Lenti-siRNA Hsp60 promote bax in mitochondria and induces apoptosis during heat stress. Biochem Biophys Res Commun. (2016) 481:125–31. doi: 10.1016/j.bbrc.2016.10.153 27818197

[63] Tang X Chang C Guo J Lincoln V Liang C Chen M. Tumour-secreted hsp90α on external surface of exosomes mediates tumour - stromal cell communication via autocrine and paracrine mechanisms. Sci Rep. (2019) 9:15108. doi: 10.1038/s41598-019-51704-w 31641193 PMC6805946

[64] Sims JD McCready J Jay DG. Extracellular heat shock protein (Hsp)70 and Hsp90α assist in matrix metalloproteinase-2 activation and breast cancer cell migration and invasion. PloS One. (2011) 6:e18848. doi: 10.1371/journal.pone.0018848 21533148 PMC3077417

[65] Sidera K Gaitanou M Stellas D Matsas R Patsavoudi E. A critical role for HSP90 in cancer cell invasion involves interaction with the extracellular domain of HER-2. J Biol Chem. (2008) 283:2031–41. doi: 10.1074/jbc.M701803200 18056992

[66] McCready J Sims JD Chan D Jay DG. Secretion of extracellular hsp90alpha via exosomes increases cancer cell motility: a role for plasminogen activation. BMC Cancer. (2010) 10:294. doi: 10.1186/1471-2407-10-294 20553606 PMC3087318

[67] Sharma MR Koltowski L Ownbey RT Tuszynski GP Sharma MC. Angiogenesis-associated protein annexin II in breast cancer: selective expression in invasive breast cancer and contribution to tumor invasion and progression. Exp Mol Pathol. (2006) 81:146–56. doi: 10.1016/j.yexmp.2006.03.003 16643892

[68] Ono K Eguchi T Sogawa C Calderwood SK Futagawa J Kasai T. HSP-enriched properties of extracellular vesicles involve survival of metastatic oral cancer cells. J Cell Biochem. (2018) 119:7350–62. doi: 10.1002/jcb.v119.9 29768689

[69] Fan CS Chen LL Hsu TA Chen CC Chua KV Li CP. Endothelial-mesenchymal transition harnesses HSP90α-secreting M2-macrophages to exacerbate pancreatic ductal adenocarcinoma. J Hematol Oncol. (2019) 12:138. doi: 10.1186/s13045-019-0826-2 31847880 PMC6918594

[70] Ono K Sogawa C Kawai H Tran MT Taha EA Lu Y. Triple knockdown of CDC37, HSP90-alpha and HSP90-beta diminishes extracellular vesicles-driven Malignancy events and macrophage M2 polarization in oral cancer. J Extracell Vesicles. (2020) 9:1769373. doi: 10.1080/20013078.2020.1769373 33144925 PMC7580842

[71] Basu S Binder RJ Suto R Anderson KM Srivastava PK. Necrotic but not apoptotic cell death releases heat shock proteins, which deliver a partial maturation signal to dendritic cells and activate the NF-kappa B pathway. Int Immunol. (2000) 12:1539–46. doi: 10.1093/intimm/12.11.1539 11058573

[72] Lv LH Wan YL Lin Y Zhang W Yang M Li GL. Anticancer drugs cause release of exosomes with heat shock proteins from human hepatocellular carcinoma cells that elicit effective natural killer cell antitumor responses *in vitro* . J Biol Chem. (2012) 287:15874–85. doi: 10.1074/jbc.M112.340588 PMC3346092 22396543

[73] Mambula SS Calderwood SK. Heat shock protein 70 is secreted from tumor cells by a nonclassical pathway involving lysosomal endosomes. J Immunol. (2006) 177:7849–57. doi: 10.4049/jimmunol.177.11.7849 17114456

[74] Kahroba H Hejazi MS Samadi N. Exosomes: from carcinogenesis and metastasis to diagnosis and treatment of gastric cancer. Cell Mol Life Sci. (2019) 76:1747–58. doi: 10.1007/s00018-019-03035-2 PMC11105779 30734835

[75] Chalmin F Ladoire S Mignot G Vincent J Bruchard M Remy-Martin JP. Membrane-associated Hsp72 from tumor-derived exosomes mediates STAT3-dependent immunosuppressive function of mouse and human myeloid-derived suppressor cells. J Clin Invest. (2010) 120:457–71. doi: 10.1172/JCI40483 PMC2810085 20093776

[76] Gross C Hansch D Gastpar R Multhoff G. Interaction of heat shock protein 70 peptide with NK cells involves the NK receptor CD94. Biol Chem. (2003) 384:267–79. doi: 10.1515/BC.2003.030 12675520

[77] Multhoff G. Heat shock protein 70 (Hsp70): membrane location, export and immunological relevance. Methods. (2007) 43:229–37. doi: 10.1016/j.ymeth.2007.06.006 17920520

[78] Asea A Kraeft SK Kurt-Jones EA Stevenson MA Chen LB Finberg RW. HSP70 stimulates cytokine production through a CD14-dependant pathway, demonstrating its dual role as a chaperone and cytokine. Nat Med. (2000) 6:435–42. doi: 10.1038/74697 10742151

[79] Panjwani NN Popova L Srivastava PK. Heat shock proteins gp96 and hsp70 activate the release of nitric oxide by APCs. J Immunol. (2002) 168:2997–3003. doi: 10.4049/jimmunol.168.6.2997 11884472

[80] Vega VL Rodríguez-Silva M Frey T Gehrmann M Diaz JC Steinem C. Hsp70 translocates into the plasma membrane after stress and is released into the extracellular environment in a membrane-associated form that activates macrophages. J Immunol. (2008) 180:4299–307. doi: 10.4049/jimmunol.180.6.4299 18322243

[81] Gastpar R Gehrmann M Bausero MA Asea A Gross C Schroeder JA. Heat shock protein 70 surface-positive tumor exosomes stimulate migratory and cytolytic activity of natural killer cells. Cancer Res. (2005) 65:5238–47. doi: 10.1158/0008-5472.CAN-04-3804 PMC1785299 15958569

[82] Multhoff G Pfister K Gehrmann M Hantschel M Gross C Hafner M. A 14-mer Hsp70 peptide stimulates natural killer (NK) cell activity. Cell Stress Chaperones. (2001) 6:337–44. doi: 10.1379/1466-1268(2001)006<0337:AMHPSN>2.0.CO;2 PMC434416 11795470

[83] Elsner L Flügge PF Lozano J Muppala V Eiz-Vesper B Demiroglu SY. The endogenous danger signals HSP70 and MICA cooperate in the activation of cytotoxic effector functions of NK cells. J Cell Mol Med. (2010) 14:992–1002. doi: 10.1111/j.1582-4934.2008.00677.x 20569278 PMC3823130

[84] Wang Y Whittall T McGowan E Younson J Kelly C Bergmeier LA. Identification of stimulating and inhibitory epitopes within the heat shock protein 70 molecule that modulate cytokine production and maturation of dendritic cells. J Immunol. (2005) 174:3306–16. doi: 10.4049/jimmunol.174.6.3306 15749862

[85] Thériault JR Mambula SS Sawamura T Stevenson MA Calderwood SK. Extracellular HSP70 binding to surface receptors present on antigen presenting cells and endothelial/epithelial cells. FEBS Lett. (2005) 579:1951–60. doi: 10.1016/j.febslet.2005.02.046 15792802

[86] Noessner E Gastpar R Milani V Brandl A Hutzler PJ Kuppner MC. Tumor-derived heat shock protein 70 peptide complexes are cross-presented by human dendritic cells. J Immunol. (2002) 169:5424–32. doi: 10.4049/jimmunol.169.10.5424 12421917

[87] Takemoto S Nishikawa M Guan X Ohno Y Yata T Takakura Y. Enhanced generation of cytotoxic T lymphocytes by heat shock protein 70 fusion proteins harboring both CD8(+) T-cell and CD4(+) T-cell epitopes. Mol Pharm. (2010) 7:1715–23. doi: 10.1021/mp1001069 20695521

[88] Rohrer KM Haug M Schwörer D Kalbacher H Holzer U. Mutations in the substrate binding site of human heat-shock protein 70 indicate specific interaction with HLA-DR outside the peptide binding groove. Immunology. (2014) 142:237–47. doi: 10.1111/imm.2014.142.issue-2 PMC4008231 24428437

[89] Figueiredo C Wittmann M Wang D Dressel R Seltsam A Blasczyk R. Heat shock protein 70 (HSP70) induces cytotoxicity of T-helper cells. Blood. (2009) 113:3008–16. doi: 10.1182/blood-2008-06-162727 19018093

[90] Wachstein J Tischer S Figueiredo C Limbourg A Falk C Immenschuh S. HSP70 enhances immunosuppressive function of CD4(+)CD25(+)FoxP3(+) T regulatory cells and cytotoxicity in CD4(+)CD25(-) T-cells. PloS One. (2012) 7:e51747. doi: 10.1371/journal.pone.0051747 23300563 PMC3530531

[91] Somensi N Brum PO de Miranda Ramos V Gasparotto J Zanotto-Filho A Rostirolla DC. Extracellular HSP70 activates ERK1/2, NF-kB and pro-inflammatory gene transcription through binding with RAGE in A549 human lung cancer cells. Cell Physiol Biochem. (2017) 42:2507–22. doi: 10.1159/000480213 28848092

[92] Evdonin AL Kropacheva IV Medvedeva ND. Extracellular Hsp70 stimulates multiple signaling pathways in A431 carcinoma cells. Biochemistry (Moscow) Supplement Series A: Membrane and Cell. Biology. (2009) 3:291–7.

[93] Odenthal J Takes R Friedl P. Plasticity of tumor cell invasion: governance by growth factors and cytokines. Carcinogenesis. (2016) 37:1117–28. doi: 10.1093/carcin/bgw098 27664164

[94] Wu FH Yuan Y Li D Liao SJ Yan B Wei JJ. Extracellular HSPA1A promotes the growth of hepatocarcinoma by augmenting tumor cell proliferation and apoptosis-resistance. Cancer Lett. (2012) 317:157–64. doi: 10.1016/j.canlet.2011.11.020 22115967

[95] Gong W Wang ZY Chen GX Liu YQ Gu XY Liu WW. Invasion potential of H22 hepatocarcinoma cells is increased by HMGB1-induced tumor NF-κB signaling via initiation of HSP70. Oncol Rep. (2013) 30:1249–56. doi: 10.3892/or.2013.2595 23836405

[96] Lee KJ Kim YM Kim DY Jeoung D Han K Lee ST. Release of heat shock protein 70 (Hsp70) and the effects of extracellular Hsp70 on matric metalloproteinase-9 expression in human monocytic U937 cells. Exp Mol Med. (2006) 38:364–74. doi: 10.1038/emm.2006.43 16953115

[97] Cappello F Czarnecka AM La Rocca G Di Stefano A Zummo G Macario AJ. Hsp60 and Hspl0 as antitumor molecular agents. Cancer Biol Ther. (2007) 6:487–9. doi: 10.4161/cbt.6.4.4087 17457039

[98] Campanella C Bucchieri F Merendino AM Fucarino A Burgio G Corona DF. The odyssey of Hsp60 from tumor cells to other destinations includes plasma membrane-associated stages and Golgi and exosomal protein-trafficking modalities. PloS One. (2012) 7:e42008. doi: 10.1371/journal.pone.0042008 22848686 PMC3405006

[99] Campanella C D’Anneo A Marino Gammazza A Caruso Bavisotto C Barone R Emanuele S. The histone deacetylase inhibitor SAHA induces HSP60 nitration and its extracellular release by exosomal vesicles in human lung-derived carcinoma cells. Oncotarget. (2016) 7:28849–67. doi: 10.18632/oncotarget.v7i20 PMC5045361 26700624

[100] Campanella C Rappa F Sciumè C Marino Gammazza A Barone R Bucchieri F. Heat shock protein 60 levels in tissue and circulating exosomes in human large bowel cancer before and after ablative surgery. Cancer. (2015) 121:3230–9. doi: 10.1002/cncr.v121.18 26060090

[101] Magner WJ Kazim AL Stewart C Romano MA Catalano G Grande C. II, and CD40 gene expression by histone deacetylase inhibitors. J Immunol. (2000) 165:7017–24. doi: 10.4049/jimmunol.165.12.7017 11120829

[102] Vinay DS Ryan EP Pawelec G Talib WH Stagg J Elkord E. Immune evasion in cancer: Mechanistic basis and therapeutic strategies. Semin Cancer Biol. (2015) 35 Suppl:S185–s98. doi: 10.1016/j.semcancer.2015.03.004 25818339

[103] Xiang Y Liu X Sun Q Liao K Liu X Zhao Z. The development of cancers research based on mitochondrial heat shock protein 90. Front Oncol. (2023) 13:1296456. doi: 10.3389/fonc.2023.1296456 38098505 PMC10720920

[104] Igarashi Y Sasada T Carvalho F. Cancer vaccines: toward the next breakthrough in cancer immunotherapy. J Immunol Res. (2020) 2020:1–13. doi: 10.1155/2020/5825401 PMC7685825 33282961

[105] Bloch O Crane CA Fuks Y Kaur R Aghi MK Berger MS. Heat-shock protein peptide complex-96 vaccination for recurrent glioblastoma: a phase II, single-arm trial. Neuro Oncol. (2014) 16:274–9. doi: 10.1093/neuonc/not203 PMC3895386 24335700

[106] Sampson JH Vlahovic G. Editorial on “heat shock protein peptide complex-96 (HSPPC-96) vaccination for recurrent glioblastoma: a phase II, single arm trial. Neuro Oncol. (2014) 16:169–70. doi: 10.1093/neuonc/not311 PMC3895395 24443362

[107] Crane CA Han SJ Ahn B Oehlke J Kivett V Fedoroff A. Individual patient-specific immunity against high-grade glioma after vaccination with autologous tumor derived peptides bound to the 96 KD chaperone protein. Clin Cancer Res. (2013) 19:205–14. doi: 10.1158/1078-0432.CCR-11-3358 22872572

[108] Shimizu Y Yoshikawa T Kojima T Shoda K Nosaka K Mizuno S. Heat shock protein 105 peptide vaccine could induce antitumor immune reactions in a phase I clinical trial. Cancer Sci. (2019) 110:3049–60. doi: 10.1111/cas.v110.10 PMC6778658 31390678

[109] Belli F Testori A Rivoltini L Maio M Andreola G Sertoli MR. Vaccination of metastatic melanoma patients with autologous tumor-derived heat shock protein gp96-peptide complexes: clinical and immunologic findings. J Clin Oncol. (2002) 20:4169–80. doi: 10.1200/JCO.2002.09.134 12377960

[110] Pilla L Patuzzo R Rivoltini L Maio M Pennacchioli E Lamaj E. A phase II trial of vaccination with autologous, tumor-derived heat-shock protein peptide complexes Gp96, in combination with GM-CSF and interferon-alpha in metastatic melanoma patients. Cancer Immunol Immunother. (2006) 55:958–68. doi: 10.1007/s00262-005-0084-8 PMC11031093 16215718

[111] Marconato L Frayssinet P Rouquet N Comazzi S Leone VF Laganga P. Randomized, placebo-controlled, double-blinded chemoimmunotherapy clinical trial in a pet dog model of diffuse large B-cell lymphoma. Clin Cancer Res. (2014) 20:668–77. doi: 10.1158/1078-0432.CCR-13-2283 24300788

[112] Testori A Richards J Whitman E Mann GB Lutzky J Camacho L. Phase III comparison of vitespen, an autologous tumor-derived heat shock protein gp96 peptide complex vaccine, with physician’s choice of treatment for stage IV melanoma: the C-100-21 Study Group. J Clin Oncol. (2008) 26:955–62. doi: 10.1200/JCO.2007.11.9941 18281670

[113] Yang JC. Vitespen: a vaccine for renal cancer? Lancet. (2008) 372:92–3. doi: 10.1016/S0140-6736(08)60698-4 18602687

[114] Wood C Srivastava P Bukowski R Lacombe L Gorelov AI Gorelov S. An adjuvant autologous therapeutic vaccine (HSPPC-96; vitespen) versus observation alone for patients at high risk of recurrence after nephrectomy for renal cell carcinoma: a multicentre, open-label, randomised phase III trial. Lancet. (2008) 372:145–54. doi: 10.1016/S0140-6736(08)60697-2 18602688

[115] Srivastava PK. Heat shock proteins in immune response to cancer: the Fourth Paradigm. Experientia. (1994) 50:1054–60. doi: 10.1007/BF01923461 7988665

[116] Hoos A Levey DL. Vaccination with heat shock protein-peptide complexes: from basic science to clinical applications. Expert Rev Vaccines. (2003) 2:369–79. doi: 10.1586/14760584 12903802

[117] Binder RJ Harris ML Ménoret A Srivastava PK. Saturation, competition, and specificity in interaction of heat shock proteins (hsp) gp96, hsp90, and hsp70 with CD11b+ cells. J Immunol. (2000) 165:2582–7. doi: 10.4049/jimmunol.165.5.2582 10946285

[118] Binder RJ Han DK Srivastava PK. CD91: a receptor for heat shock protein gp96. Nat Immunol. (2000) 1:151–5. doi: 10.1038/77835 11248808

[119] Srivastava PK Maki RG. Stress-induced proteins in immune response to cancer. Curr Top Microbiol Immunol. (1991) 167:109–23. doi: 10.1073/pnas.91.8.3077 1711433

[120] Singh-Jasuja H Scherer HU Hilf N Arnold-Schild D Rammensee HG Toes RE. The heat shock protein gp96 induces maturation of dendritic cells and down-regulation of its receptor. Eur J Immunol. (2000) 30:2211–5. doi: 10.1002/1521-4141(2000)30:8<2211::AID-IMMU2211>3.0.CO;2-0 10940912

[121] Hilf N Singh-Jasuja H Schwarzmaier P Gouttefangeas C Rammensee HG Schild H. Human platelets express heat shock protein receptors and regulate dendritic cell maturation. Blood. (2002) 99:3676–82. doi: 10.1182/blood.V99.10.3676 11986223

[122] Udono H Levey DL Srivastava PK. Cellular requirements for tumor-specific immunity elicited by heat shock proteins: tumor rejection antigen gp96 primes CD8+ T-cells *in vivo* . Proc Natl Acad Sci U.S.A. (1994) 91:3077–81. doi: 10.1073/pnas.91.8.3077 PMC43518 7909157

[123] Suto R Srivastava PK. A mechanism for the specific immunogenicity of heat shock protein-chaperoned peptides. Science. (1995) 269:1585–8. doi: 10.1126/science.7545313 7545313

[124] Udono H Srivastava PK. Heat shock protein 70-associated peptides elicit specific cancer immunity. J Exp Med. (1993) 178:1391–6. doi: 10.1084/jem.178.4.1391 PMC2191193 8376942

[125] Weng D Calderwood SK Gong J. A novel heat shock protein 70-based vaccine prepared from DC-tumor fusion cells. Methods Mol Biol. (2018) 1709:359–69. doi: 10.1007/978-1-4939-7477-1_26 29177672

[126] Enomoto Y Bharti A Khaleque AA Song B Liu C Apostolopoulos V. Enhanced immunogenicity of heat shock protein 70 peptide complexes from dendritic cell-tumor fusion cells. J Immunol. (2006) 177:5946–55. doi: 10.4049/jimmunol.177.9.5946 17056519

[127] Chen Y Zheng L Hua W Wang J Chen L Huang A. Fusion of NY-ESO-1 epitope with heat shock protein 70 enhances its induced immune responses and antitumor activity against glioma *in vitro* . Transl Cancer Res. (2024) 13:191–201. doi: 10.21037/tcr-23-1476 38410235 PMC10894325

[128] Choi DH Woo JK Choi Y Seo HS Kim CW. A novel chimeric DNA vaccine: enhancement of preventive and therapeutic efficacy of DNA vaccine by fusion of Mucin 1 to a heat shock protein 70 gene. Mol Med Rep. (2011) 4:885–90. doi: 10.3892/mmr.2011.525 21725596

[129] Ma JH Sui YF Ye J Huang YY Li ZS Chen GS. Heat shock protein 70/MAGE-3 fusion protein vaccine can enhance cellular and humoral immune responses to MAGE-3 *in vivo* . Cancer Immunol Immunother. (2005) 54:907–14. doi: 10.1007/s00262-004-0660-3 PMC11034288 15756604

[130] Zhang H Wang S Sun M Cui Y Xing J Teng L. Exosomes as smart drug delivery vehicles for cancer immunotherapy. Front Immunol. (2022) 13:1093607. doi: 10.3389/fimmu.2022.1093607 36733388 PMC9888251

[131] Abdul NS Ahmad Alrashed N Alsubaie S Albluwi H Badr Alsaleh H Alageel N. Role of extracellular heat shock protein 90 alpha in the metastasis of oral squamous cell carcinoma: A systematic review. Cureus. (2023) 15:e38514. doi: 10.7759/cureus.38514 37273315 PMC10238764

[132] Linderoth NA Popowicz A Sastry S. Identification of the peptide-binding site in the heat shock chaperone/tumor rejection antigen gp96 (Grp94). J Biol Chem. (2000) 275:5472–7. doi: 10.1074/jbc.275.8.5472 10681525

[133] Castelli C Rivoltini L Rini F Belli F Testori A Maio M. Heat shock proteins: biological functions and clinical application as personalized vaccines for human cancer. Cancer Immunol Immunother. (2004) 53:227–33. doi: 10.1007/s00262-003-0481-9 PMC11034345 14689240

[134] Mellman I Steinman RM. Dendritic cells: specialized and regulated antigen processing machines. Cell. (2001) 106:255–8. doi: 10.1016/S0092-8674(01)00449-4 11509172

[135] Naseri M Bozorgmehr M Zöller M Ranaei Pirmardan E Madjd Z. Tumor-derived exosomes: the next generation of promising cell-free vaccines in cancer immunotherapy. Oncoimmunology. (2020) 9:1779991. doi: 10.1080/2162402X.2020.1779991 32934883 PMC7466856

[136] Feng H Zeng Y Whitesell L Katsanis E. Stressed apoptotic tumor cells express heat shock proteins and elicit tumor-specific immunity. Blood. (2001) 97:3505–12. doi: 10.1182/blood.V97.11.3505 11369644

[137] Zhou J Kroll AV Holay M Fang RH Zhang L. Biomimetic nanotechnology toward personalized vaccines. Adv Mater. (2020) 32:e1901255. doi: 10.1002/adma.201901255 31206841 PMC6918015

[138] Chen W Wang J Shao C Liu S Yu Y Wang Q. Efficient induction of antitumor T-cell immunity by exosomes derived from heat-shocked lymphoma cells. Eur J Immunol. (2006) 36:1598–607. doi: 10.1002/eji.200535501 16708399

[139] Menay F Herschlik L De Toro J Cocozza F Tsacalian R Gravisaco MJ. Exosomes isolated from ascites of T-cell lymphoma-bearing mice expressing surface CD24 and HSP-90 induce a tumor-specific immune response. Front Immunol. (2017) 8:286. doi: 10.3389/fimmu.2017.00286 28360912 PMC5352668

[140] Behzadi E Hosseini HM Halabian R Fooladi AAI. Macrophage cell-derived exosomes/staphylococcal enterotoxin B against fibrosarcoma tumor. Microb Pathog. (2017) 111:132–8. doi: 10.1016/j.micpath.2017.08.027 28843722

[141] Day NB Wixson WC Shields C. Magnetic systems for cancer immunotherapy. Acta Pharm Sin B. (2021) 11:2172–96. doi: 10.1016/j.apsb.2021.03.023 PMC8424374 34522583

[142] Sen K Sheppe AEF Singh I Hui WW Edelmann MJ Rinaldi C. Exosomes released by breast cancer cells under mild hyperthermic stress possess immunogenic potential and modulate polarization *in vitro* in macrophages. Int J Hyperthermia. (2020) 37:696–710. doi: 10.1080/02656736.2020.1778800 32568583 PMC8694666

[143] Li X Cai X Zhang Z Ding Y Ma R Huang F. Mimetic heat shock protein mediated immune process to enhance cancer immunotherapy. Nano Lett. (2020) 20:4454–63. doi: 10.1021/acs.nanolett.0c01230 32401534

[144] Zhou Y Wang L Chen L Wu W Yang Z Wang Y. Glioblastoma cell-derived exosomes functionalized with peptides as efficient nanocarriers for synergistic chemotherapy of glioblastoma with improved biosafety. Nano Res. (2023) 16:13283–93. doi: 10.1007/s12274-023-5921-6

[145] Chen T Guo J Yang M Zhu X Cao X. Chemokine-containing exosomes are released from heat-stressed tumor cells via lipid raft-dependent pathway and act as efficient tumor vaccine. J Immunol (Baltimore Md: 1950). (2011) 186:2219–28. doi: 10.4049/jimmunol.1002991 21242526

[146] Guo D Chen Y Wang S Yu L Shen Y Zhong H. Exosomes from heat-stressed tumour cells inhibit tumour growth by converting regulatory T-cells to Th17 cells via IL-6. Immunology. (2018) 154:132–43. doi: 10.1111/imm.2018.154.issue-1 PMC5904701 29197065

[147] Liang K Sun Y Xie L Liu Y You Y Xu J. Biologically self-assembled tumor cell-derived cancer nanovaccines as an all-in-one platform for cancer immunotherapy. ACS Nano. (2024) 18:6702–17. doi: 10.1021/acsnano.4c01050 38359389

[148] Martinvalet D. Mitochondrial entry of cytotoxic proteases: A new insight into the granzyme B cell death pathway. Oxid Med Cell longevity. (2019) 2019:9165214. doi: 10.1155/2019/9165214 PMC6556269 31249651

[149] Rezaei F Bolhassani A Sadat SM Arashkia A Fotouhi F Milani A. Development of novel HPV therapeutic vaccine constructs based on engineered exosomes and tumor cell lysates. Life Sci. (2024) 340:122456. doi: 10.1016/j.lfs.2024.122456 38266814

[150] Peinado H Alečković M Lavotshkin S Matei I Costa-Silva B Moreno-Bueno G. Melanoma exosomes educate bone marrow progenitor cells toward a pro-metastatic phenotype through MET. Nat Med. (2012) 18:883–91. doi: 10.1038/nm.2753 PMC3645291 22635005

[151] Caruso Bavisotto C Cipolla C Graceffa G Barone R Bucchieri F Bulone D. Immunomorphological pattern of molecular chaperones in normal and pathological thyroid tissues and circulating exosomes: potential use in clinics. Int J Mol Sci. (2019) 20(18):4496. doi: 10.3390/ijms20184496 31514388 PMC6770414

[152] Albakova Z Siam MKS Sacitharan PK Ziganshin RH Ryazantsev DY Sapozhnikov AM. Extracellular heat shock proteins and cancer: New perspectives. Transl Oncol. (2021) 14:100995. doi: 10.1016/j.tranon.2020.100995 33338880 PMC7749402

[153] Liu W Li J Zhang P Hou Q Feng S Liu L. A novel pan-cancer biomarker plasma heat shock protein 90alpha and its diagnosis determinants in clinic. Cancer Sci. (2019) 110:2941–59. doi: 10.1111/cas.v110.9 PMC6726694 31343810

[154] Chanteloup G Cordonnier M Isambert N Bertaut A Hervieu A Hennequin A. Monitoring HSP70 exosomes in cancer patients’ follow up: a clinical prospective pilot study. J Extracell Vesicles. (2020) 9:1766192. doi: 10.1080/20013078.2020.1766192 32595915 PMC7301715

[155] Tang T Yang C Brown HE Huang J. Circulating heat shock protein 70 is a novel biomarker for early diagnosis of lung cancer. Dis Markers. (2018) 2018:6184162. doi: 10.1155/2018/6184162 30245753 PMC6136573

[156] Gold B Cankovic M Furtado LV Meier F Gocke CD. Do circulating tumor cells, exosomes, and circulating tumor nucleic acids have clinical utility? A report of the association for molecular pathology. J Mol Diagn. (2015) 17:209–24. doi: 10.1016/j.jmoldx.2015.02.001 PMC4411248 25908243

[157] Kulasingam V Prassas I Diamandis EP. Towards personalized tumor markers. NPJ Precis Oncol. (2017) 1:17. doi: 10.1038/s41698-017-0021-2 29872704 PMC5871887

[158] Elhendawy HA. Clinical implications of heat shock protein 70 in oral carcinogenesis and prediction of progression and recurrence in oral squamous cell carcinoma patients: a retrospective clinicopathological study. Eur J Med Res. (2023) 28:464. doi: 10.1186/s40001-023-01433-8 37884988 PMC10604814

[159] Ostheimer C Gunther S Bache M Vordermark D Multhoff G. Dynamics of heat shock protein 70 serum levels as a predictor of clinical response in non-small-cell lung cancer and correlation with the hypoxia-related marker osteopontin. Front Immunol. (2017) 8:1305. doi: 10.3389/fimmu.2017.01305 29093708 PMC5651249

[160] Lee HW Lee EH Kim SH Roh MS Jung SB Choi YC. Heat shock protein 70 (HSP70) expression is associated with poor prognosis in intestinal type gastric cancer. Virchows Arch. (2013) 463:489–95. doi: 10.1007/s00428-013-1461-x 23913168

[161] Mano R Zilber S Di Natale RG Kedar D Lifshitz DA Yossepowitch O. Heat shock proteins 60 and 70 are associated with long-term outcome of T1-stage high-grade urothelial tumors of the bladder treated with intravesical Bacillus Calmette-Guérin immunotherapy. Urol Oncol. (2018) 36:531.e9–.e17. doi: 10.1016/j.urolonc.2018.09.007 PMC6289697 30337218

[162] Lennartz P Thölke D Bashiri Dezfouli A Pilz M Lobinger D Messner V. Biomarkers in adult-type diffuse gliomas: elevated levels of circulating vesicular heat shock protein 70 serve as a biomarker in grade 4 glioblastoma and increase NK cell frequencies in grade 3 glioma. Biomedicines. (2023) 11(12):3235. doi: 10.3390/biomedicines11123235 38137456 PMC10741018

[163] Meng Q Li BX Xiao X. Toward developing chemical modulators of hsp60 as potential therapeutics. Front Mol Biosci. (2018) 5:35. doi: 10.3389/fmolb.2018.00035 29732373 PMC5920047

