# Corrigendum: A new weapon: the application of tumor vaccines based on extracellular exosomal heat shock proteins in immunotherapy

**DOI:** 10.3389/fimmu.2025.1610622

**Published:** 2025-04-25

**Authors:** Kexin Yi, Chengpeng Sun, Yalin Yuan, Zhaowei Luo, Hongliang Luo, Yunhe Xie

**Affiliations:** ^1^ Department of General Surgery, Jiujiang Hospital of Traditional Chinese Medicine, Jiujiang, Jiangxi, China; ^2^ The Second Clinical Medical College, Nanchang University, Nanchang, China; ^3^ Huankui Academy, Jiangxi Medical College, Nanchang University, Nanchang, Jiangxi, China; ^4^ Department of General Surgery, The Second Affiliated Hospital, Jiangxi Medical College, Nanchang University, Nanchang, Jiangxi, China

**Keywords:** heat shock proteins, exosomes, immunotherapy, tumor vaccines, tumor microenvironment

In the published article, there was an error in affiliation(s) 1-4. Instead of “4”, it should be “1”. Instead of “2”, it should be “3”. Instead of “3”, it should be “4”. Instead of “1”, it should be “2”.

In the published article, there was also an error regarding the affiliation(s) for Kexin Yi. As well as having affiliation(s) 2(s), they should also have “Department of General Surgery, Jiujiang Hospital of Traditional Chinese Medicine, Jiujiang, Jiangxi, China”.

The authors apologize for this error and state that this does not change the scientific conclusions of the article in any way. The original article has been updated.

